# New substituted quinoxalines inhibit triple-negative breast cancer by specifically downregulating the *c-MYC* transcription

**DOI:** 10.1093/nar/gkz835

**Published:** 2019-10-04

**Authors:** Ming-Hao Hu, Tian-Ying Wu, Qiong Huang, Guangyi Jin

**Affiliations:** 1 School of Pharmaceutical Sciences, Shenzhen University Health Science Center, Shenzhen 518060, China; 2 International Cancer Center, Shenzhen University Health Science Center, Shenzhen 518060, China

## Abstract

Conventional chemotherapy remains the primary treatment option for triple-negative breast cancer (TNBC). However, the current chemotherapeutic drugs have limited effects on TNBC, and often lead to serious side effects as well as drug resistance. Thus, more effective therapeutic options are sorely needed. As *c-MYC* oncogene is highly expressed during TNBC pathogenesis, inhibiting *c-MYC* expression would be an alternative anti-TNBC strategy. In this study, we designed and synthesized a serial of quinoxaline analogs that target *c-MYC* promoter G-quadruplex (G4), which is believed to be a repressor of *c-MYC* transcription. Among them, a difluoro-substituted quinoxaline **QN-1** was identified as the most promising G4-stabilizing ligand with high selectivity to *c-MYC* G4 over other G4s, which is distinguished from many other reported ligands. Intracellular studies indicated that **QN-1** induced cell cycle arrest and apoptosis, repressed metastasis and inhibited TNBC cell growth, primarily due to the downregulation of *c-MYC* transcription by a G4-dependent mechanism. Notably, inhibition by **QN-1** was significantly greater for *c-MYC* than other G4-driven genes. Cancer cells with c-MYC overexpression were more sensitive to **QN-1**, relative to normal cells. Furthermore, **QN-1** effectively suppressed tumor growth in a TNBC mouse model. Accordingly, this work provides an alternative strategy for treating TNBC.

## INTRODUCTION

Triple-negative breast cancer (TNBC) is a subtype of breast cancer with an aggressive phenotype which shows high metastatic capability and poor prognosis. TNBC accounts for 10–20% of diagnosed breast cancers and is characterized by the negative expression of progesterone receptor (PR), estrogen receptor (ER), and human epidermal growth factor receptor 2 (HER2) (1). Consequently, convenient targeted therapies used for the hormone receptor-positive breast cancers that target these receptors (e.g. tamoxifen, lapatinib or trastuzumab) are not effective for TNBC, which leaves cytotoxic chemotherapy as a mainstay for the treatment of TNBC (2–4). However, the current chemotherapeutic drugs, such as paclitaxel, cisplatin and doxorubicin, have limited effects on TNBC as well as serious side effects. Besides, high-dose chemotherapy often leads to disease relapse and drug resistance (5,6). Hence, despite comprehensive management, over 50% of TNBC patients recur, and more than 37% of those patients succumb within 5 years (2). Recently, PARP inhibitors (olaparib and talazoparib) were approved to treat HER2-negative breast cancer with an inherited BRCA1 or BRCA2 mutation (7,8). However, mutations in BRCA account for only 10–20% of TNBC (8). Moreover, these drugs might also cause serious side effects (e.g. myelodysplastic syndrome, acute myeloid leukemia). Therefore, it is of great importance to identify more effective agents with fewer side effects for the treatment of TNBC.

The *c-MYC* gene is well known as an important oncogene that plays a crucial role in cell metabolism, growth, proliferation, differentiation and apoptosis (9,10). Elevated c-MYC expression are observed in 80% of human cancer cells, including TNBC, which promotes tumorigenesis (11). Notably, c-MYC overexpression is closely related to the development, metastasis and drug resistance of TNBC, leading to poor clinical prognosis (11–15). It is worth noting that high level of c-MYC expression results in a significant increase in cancer stem-like cells (CSCs) (16), and drives metabolic reprogramming in TNBC (17,18). Therefore, inhibition of c-MYC would be an effective strategy for treating TNBC (16,19,20). However, the identification of inhibitors directly targeting c-MYC protein seems to be challenging given the absence of a well-defined ligand-binding pocket (11). Thus, downregulation of the *c-MYC* gene should be an alternative approach to the treatment of TNBC, but few studies have focused on it.

As is known, the nuclease hypersensitive element III_1_ (NHE III_1_), located upstream of the P1 promoter, governs the *c-MYC* transcription. Notably, this region contains a guanine-rich sequence that can fold into a specific DNA secondary structure, known as the G-quadruplex (G4), which is likely to act as a transcription repressor (21). Stabilization of this G4 structure by small-molecule ligands would lead to downregulation of the *c-MYC* transcription, which has developed into a new anticancer drug discovery strategy (22). Hence, ligands that stabilize the *c-MYC* G4 might also act as effective agents for TNBC treatment. Various small molecules have been synthesized and tested for their abilities to stabilize the *c-MYC* G4, including quindolines (23–25), berberines (26), porphyrins (27,28), imidazoles (29) and others (30). Although the planar and aromatic scaffold of these molecules provides good recognition for G4 through π−π stacking interactions, such structures exhibit poor solubility, high molecular weights, or multiple cationic charges, falling outside ‘drug-like’ chemical space. Furthermore, few ligands show good selectivity between the *c-MYC* G4 and other G4s (31,32). As a growing number of G4-driven biological events have been reported, the expanded variety of G4 ligands that possess differential binding profiles is becoming more and more important, which might also display fewer side effects.

In this study, we reported the discovery of a drug-like compound with dramatic inhibitory effects on TNBC, and demonstrated that it inhibited the *c-MYC* transcription by a G4-dependent mechanism. First, we designed and synthesized a small library of quinoxaline analogs, which were evaluated for their affinities to the *c-MYC* G4, and their abilities to inhibit cell growth of TNBC. Among them, **QN-1** was identified as the most promising ligand. Then, the detailed interactions of **QN-1** with the *c-MYC* G4 were studied using various experiments, including absorption titrations, CD assays, NMR titrations and 2-Ap fluorescent titrations. Furthermore, we demonstrated that **QN-1** downregulated the *c-MYC* transcription by targeting its promoter G4 via RT-PCR, Western blotting and CA46 exon-specific assay. Importantly, it did not downregulate several other G4-dependent genes to the same extent. Furthermore, the anticancer activity of **QN-1** was confirmed in a TNBC mouse model.

## MATERIALS AND METHODS

### Synthesis and characterization

^1^H and ^13^C NMR spectra were recorded by using TMS as the internal standard in CDCl_3_ at 600 MHz and 151 MHz, with a Bruker BioSpin GmbH spectrometer. High resolution mass spectra (HRMS) were recorded on a SCIEX TripleTOF 6600. Flash column chromatography was performed with silica gel (200–300 mesh) purchased from Qingdao Haiyang Chemical Co. Ltd. The purity of the synthesized compounds were confirmed to be higher than 95% by using analytical HPLC. The NMR and HRMS spetra of the final compounds were provided in the Supplementary Figures S17–S46.

The intermediates **2** and **3** were synthesized according to our previous study (33). The general method for synthesis of **QN-1**–**QN-10** is described below: A mixture of compound **2** (or **3**) (1.0 mmol), *o*-phenylenediamine derivative (2.0 mmol), AcOH (3 drops) and EtOH (10 ml) was stirred at reflux temperature for 24 h. After cooling, the precipitate was filtered, and then washed with EtOH to get the pure product (**QN-1, QN-2, QN-4** and **QN-10**). If there was no precipitate, the solvent was evaporated to get the crude product, and then it was purified using flash column chromatography to obtain the final product (**QN-3, QN-7, QN-8** and **QN-9**). To prepare **QN-5** and **QN-6**, the starting materials, including **QN-1** (1.0 mmol), *N*-methyl piperazine or morpholine (5.0 mmol) and K_2_CO_3_ (1.0 mmol), were dispersed and stirred in DMSO under 90°C overnight. After cooling, the mixture was poured into cold water, and the precipitate was filtered to get the final compounds.

#### 6,7-Difluoro-2,3-bis(4-(4-methylpiperazin-1-yl)phenyl)quinoxaline (**QN-1**)

Yellow solid (68% yield). ^1^H NMR (500 MHz, CDCl_3_) δ 7.81 (t, *J* = 9.4 Hz, 2H), 7.48 (d, *J* = 8.8 Hz, 4H), 6.87 (d, *J* = 8.9 Hz, 4H), 3.42–3.20 (m, 8H), 2.68–2.52 (m, 8H), 2.36 (s, 6H). ^13^C NMR (126 MHz, CDCl_3_) δ 153.33, 152.08, 151.53, 138.14, 130.79, 129.44, 114.98, 114.48, 54.92, 48.21, 46.12. HRMS (ESI) *m*/*z*: calcd for C_30_H_32_F_2_N_6_: 515.2729 [M+H]^+^, found 515.2719 [M+H]^+^.

#### 2,3-bis(4-(4-Methylpiperazin-1-yl)phenyl)quinoxaline (**QN-2**)

Yellow solid (75% yield). ^1^H NMR (600 MHz, CDCl_3_) δ 8.13–8.08 (m, 2H), 7.72–7.67 (m, 2H), 7.52 (d, *J* = 8.8 Hz, 4H), 6.89 (d, *J* = 8.8 Hz, 4H), 3.35–3.26 (m, 8H), 2.64–2.56 (m, 8H), 2.38 (s, 6H). ^13^C NMR (151 MHz, CDCl_3_) δ 153.17, 151.36, 141.02, 130.84, 130.19, 129.18, 128.92, 115.11, 54.96, 48.37, 46.15. HRMS (ESI) *m*/*z*: calcd for C_30_H_34_N_6_: 479.2918 [M+H]^+^, found 479.2911 [M+H]^+^.

#### 6-Methoxy-2,3-bis(4-(4-methylpiperazin-1-yl)phenyl)quinoxaline (**QN-3**)

Yellow solid (43% yield). ^1^H NMR (600 MHz, CDCl_3_) δ 7.99 (d, *J* = 9.1 Hz, 1H), 7.51–7.46 (m, 4H), 7.42 (d, *J* = 2.7 Hz, 1H), 7.38–7.33 (m, 1H), 6.91–6.86 (m, 4H), 3.98 (s, 3H), 3.34–3.24 (m, 8H), 2.65–2.56 (m, 8H), 2.38 (s, 6H). ^13^C NMR (151 MHz, CDCl_3_) δ 160.35, 153.06, 151.27, 151.10, 150.76, 142.46, 137.09, 130.80, 130.70, 130.48, 130.34, 129.90, 122.38, 115.22, 115.13, 106.44, 55.78, 54.96, 54.95, 48.48, 48.39, 46.13. HRMS (ESI) *m*/*z*: calcd for C_31_H_36_N_6_O: 509.3023 [M+H]^+^, found 509.3022 [M+H]^+^.

#### 2,3-bis(4-(4-Methylpiperazin-1-yl)phenyl)-6-(trifluoromethyl)quinoxaline (**QN-4**)

Brick-red solid (77% yield). ^1^H NMR (600 MHz, CDCl_3_) δ 8.41 (s, 1H), 8.19 (d, *J* = 8.7 Hz, 1H), 7.85 (dd, *J* = 8.7, 1.9 Hz, 1H), 7.57–7.52 (m, 4H), 6.92–6.88 (m, 4H), 3.36–3.27 (m, 8H), 2.65–2.57 (m, 8H), 2.39 (s, 6H). ^13^C NMR (151 MHz, CDCl_3_) δ 155.00, 154.44, 151.73, 151.66, 142.04, 139.90, 130.96, 130.89, 130.63, 129.98, 129.32, 126.90, 124.70, 114.92, 54.89, 48.11, 46.11. HRMS (ESI) *m*/*z*: calcd for C_31_H_33_F_3_N_6_: 547.2792 [M+H]^+^, found 547.2792 [M+H]^+^.

#### 6-Fluoro-7-(4-methylpiperazin-1-yl)-2,3-bis(4-(4-methylpiperazin-1-yl)phenyl)quinoxaline (**QN-5**)

Yellow solid (70% yield). ^1^H NMR (600 MHz, CDCl_3_) δ 7.67 (d, *J* = 13.3 Hz, 1H), 7.50 (d, *J* = 8.9 Hz, 1H), 7.48–7.42 (m, 4H), 6.90–6.85 (m, 4H), 3.39–3.31 (m, 4H), 3.29–3.25 (m, 8H), 2.76–2.63 (m, 4H), 2.63–2.56 (m, 8H), 2.41 (s, 3H), 2.37 (s, 6H). ^13^C NMR (151 MHz, CDCl_3_) δ 157.37, 152.49, 151.49, 151.25, 151.21, 143.37, 139.24, 137.60, 130.74, 130.69, 130.31, 130.15, 115.41, 115.17, 115.11, 113.06, 54.99, 54.95, 50.54, 50.51, 48.42, 48.40, 46.14. HRMS (ESI) m/z: calcd for C_35_H_43_FN_8_: 595.3667 [M+H]^+^, found 595.3657 [M+H]^+^.

#### 4-(7-fluoro-2,3-bis(4-(4-methylpiperazin-1-yl)phenyl)quinoxalin-6-yl)morpholine (**QN-6**)

Yellow solid (67% yield). ^1^H NMR (600 MHz, CDCl_3_) δ 7.68 (d, *J* = 13.2 Hz, 1H), 7.51–7.44 (m, 5H), 6.92–6.83 (m, 4H), 4.00–3.90 (m, 4H), 3.36–3.20 (m, 12H), 2.64–2.52 (m, 4H), 2.37 (s, 6H). ^13^C NMR (151 MHz, CDCl_3_) δ 157.26, 152.58, 151.69, 151.29, 151.26, 143.18, 139.19, 137.64, 130.75, 130.69, 130.21, 130.05, 115.27, 115.14, 115.09, 113.18, 66.85, 54.96, 50.97, 50.95, 48.41, 48.39, 46.16. HRMS (ESI) *m*/*z*: calcd for C_34_H_40_FN_7_O: 582.3351 [M+H]^+^, found 582.3341 [M+H]^+^.

#### 6,7-Difluoro-2-(4-fluorophenyl)-3-(4-(4-methylpiperazin-1-yl)phenyl)quinoxaline (**QN-7**)

Yellow solid (65% yield). ^1^H NMR (600 MHz, CDCl_3_) δ 7.86 (ddd, *J* = 10.4, 8.2, 5.6 Hz, 2H), 7.59–7.53 (m, 2H), 7.43 (d, *J* = 8.8 Hz, 2H), 7.11–7.04 (m, 2H), 6.87 (d, *J* = 8.9 Hz, 2H), 3.38–3.29 (m, 4H), 2.72–2.61 (m, 4H), 2.41 (s, 3H). ^13^C NMR (151 MHz, CDCl_3_) δ 163.19, 153.18, 153.12, 152.49, 151.53, 151.43, 138.65, 137.91, 135.10, 131.62, 130.97, 128.68, 115.54, 114.99, 114.63, 114.58, 54.54, 47.77, 45.70. HRMS (ESI) *m*/*z*: calcd for C_25_H_21_F_3_N_4_: 435.1791 [M+H]^+^, found 435.1792 [M+H]^+^.

#### 6-Chloro-2,3-bis(4-(4-methylpiperazin-1-yl)phenyl)pyrido[2,3-b]pyrazine (**QN-8**)

Brown solid (45% yield). ^1^H NMR (600 MHz, CDCl_3_) δ 8.33 (d, *J* = 8.6 Hz, 1H), 7.66 (d, *J* = 8.9 Hz, 2H), 7.59 (d, *J* = 8.6 Hz, 1H), 7.55 (d, *J* = 8.8 Hz, 2H), 6.90 (d, *J* = 8.9 Hz, 2H), 6.84 (d, *J* = 9.0 Hz, 2H), 3.39–3.31 (m, 8H), 2.71–2.61 (m, 8H), 2.41 (s, 3H), 2.41 (s, 3H). ^13^C NMR (151 MHz, CDCl_3_) δ 156.36, 154.37, 153.13, 151.79, 151.67, 149.14, 139.90, 134.63, 131.58, 130.81, 129.26, 128.29, 125.83, 115.05, 114.41, 54.74, 47.91, 47.57, 45.90. HRMS (ESI) *m*/*z*: calcd for C_29_H_32_ClN_7_: 514.2480 [M+H]^+^, found 514.2469 [M+H]^+^.

#### 4-((2,3-bis(4-(4-Methylpiperazin-1-yl)phenyl)quinoxalin-6-yl)oxy)aniline (**QN-9**)

Yellow solid (50% yield). ^1^H NMR (600 MHz, CDCl_3_) δ 8.04 (d, *J* = 9.1 Hz, 1H), 7.53–7.41 (m, 5H), 7.35 (d, *J* = 2.6 Hz, 1H), 6.99 (d, *J* = 8.7 Hz, 2H), 6.93–6.80 (m, 4H), 6.73 (d, *J* = 8.7 Hz, 2H), 3.33–3.23 (m, 8H), 2.66–2.55 (m, 8H), 2.38 (s, 3H), 2.37 (s, 3H). ^13^C NMR (151 MHz, CDCl_3_) δ 160.20, 153.28, 151.26, 151.21, 151.13, 147.41, 143.61, 142.07, 137.41, 130.77, 130.71, 130.40, 130.27, 130.16, 122.27, 121.97, 116.36, 115.20, 115.11, 111.30, 54.88, 54.86, 48.38, 48.31, 46.04. HRMS (ESI) *m*/*z*: calcd for C_36_H_39_N_7_O: 586.3289 [M+H]^+^, found 586.3280 [M+H]^+^.

#### 2,3-bis(4-(4-Methylpiperazin-1-yl)phenyl)benzo[g]quinoxaline (**QN-10**)

Brick-red solid (85% yield). ^1^H NMR (600 MHz, CDCl_3_) δ 8.66 (s, 2H), 8.09 (dd, *J* = 6.4, 3.2 Hz, 2H), 7.58 (d, *J* = 8.7 Hz, 4H), 7.54 (dd, *J* = 6.5, 3.1 Hz, 2H), 6.91 (d, *J* = 8.8 Hz, 4H), 3.37–3.25 (m, 8H), 2.67–2.55 (m, 8H), 2.39 (s, 6H). ^13^C NMR (151 MHz, CDCl_3_) δ 154.00, 151.54, 138.05, 133.73, 130.95, 130.17, 128.46, 126.96, 126.26, 114.95, 54.95, 48.28, 46.16. HRMS (ESI) *m*/*z*: calcd for C_34_H_36_N_6_: 529.3074 [M+H]^+^, found 529.3059 [M+H]^+^.

### Materials

All oligonucleotides (Supplementary Table S1) were dissolved in Tris–HCl buffer, and their concentrations were determined based on absorbance at 260 nm using a NanoDrop 1000 spectrophotometer (Thermo Scientific). To obtain G4 structures, oligonucleotides were annealed in relevant buffers containing 100 mM KCl by heating at 95°C for 5 min, followed by gradual cooling to room temperature. G4 formation was determined by circular dichroism (CD) spectrophotometer. Stock solutions of compounds (10 mM) were dissolved in DMSO and stored at −80°C. Further dilutions to the working concentrations were performed with the relevant buffer immediately prior to use.

### UV–vis absorption spectroscopy

UV–vis absorption studies were performed on an Agilent Cary 60 UV-Vis Spectrophotometer (Agilent Technologies) using 1 cm path length quartz cuvette. For the titration experiments, small aliquots of a stock oligonucleotide solution were added into the solution containing **QN-1** at a fixed concentration in Tris–HCl buffer. After each addition, the reaction was stirred and allowed to equilibrate for 1 min and its absorbance measurement was taken. Then, the titration data were fitted to the Benesi–Hildebrand equation (linear regression) (34):

$$\begin{equation*}\ \frac{1}{{A - {A_0}}} = \frac{1}{{{K_{\rm a}}\left( {{A_{{\rm max}}} - {A_0}} \right)\left[ {{\rm DNA}} \right]}}\ + \frac{1}{{{A_{{\rm max}}} - {A_0}}}\end{equation*}$$

where *A* is the experimentally measured absorption intensity, *A*_0_ is the absorption intensity of free **QN-1**, and *A*_max_ is the saturated absorption intensity of the **QN-1**/DNA complex. The association constant (*K*_a_) was evaluated by plotting 1/[*A* – *A*_0_] versus 1/[DNA].

### SPR study

SPR was performed on a ProteOn XPR36 Protein Interaction Array system (Bio-Rad Laboratories, CA) using a streptavidin-coated GLH sensor chip. Biotinylated pu27 was attached to the chip. Five concentrations of **QN-1** were injected simultaneously at a flow rate of 50 μl/min for 400 s of association phase, followed by 500 s of dissociation phase at 25°C. The data were analyzed with ProteOn manager software, using the Langmuir model for fitting data.

### Circular dichroism spectroscopy

CD studies were performed on a Chirascan circular dichroism spectrophotometer (Applied Photophysics). A quartz cuvette with a 10 mm path length was used to record the spectra over a wavelength range of 230–330 nm with a 1 nm bandwidth, 1 nm step size and a time of 0.5 s per point. The DNA samples were set at a concentration of 2 μM. CD melting assays were performed at a fixed G4 concentration (2 μM), either with or without a fixed concentration (2 μM) of **QN-1** in Tris−HCl buffer with 0.5 mM KCl. The data were recorded at intervals of 5°C over a range of 25–95°C with a heating rate of 1°C/min. The final analysis of the data was conducted using Origin 9.0.

### NMR titration

G4 samples for 1D NMR were prepared in phosphate buffer (25 mM KH_2_PO_4_, 70 mM KCl, 10% D_2_O, pH 7.2). The final concentration of G4 DNA was 200 μM, titrated with increasing amounts of **QN-1**. Experiments were performed on a 600 MHz spectrometer (Bruker) at 25°C.

### Cell cycle analysis

4T1 cells (3 × 10^5^ cells per well in a six-well plate) treated with **QN-1** at various concentrations were harvested and washed in PBS and fixed with 70% ethanol at 4°C overnight. Then, the cells were centrifuged and resuspended in a staining solution (50 μg/ml propidium iodide (PI), 75 KU/ml RNase A in PBS) for 30 min at room temperature in the dark. The cells were analyzed by flow cytometry using an Attune NxT Flow Cytometer (Thermo Fisher Scientific). For each analysis, 2 × 10^4^ events were collected.

### Apoptosis analysis

4T1 cells (3 × 10^5^ cells per well in a six-well plate) treated with **QN-1** at various concentrations were harvested and washed in PBS. Then, they were centrifuged and resuspended in Annexin-binding buffer. After that, the cells were incubated with Annexin V–iFluor™ 633 and PI for 15 min at room temperature in the dark and immediately analyzed by flow cytometry using an Attune NxT Flow Cytometer (Thermo Fisher Scientific). For each analysis, 2 × 10^4^ events were collected. The data are presented as bi-parametric dot plots showing PI fluorescence against Annexin V–iFluor™ 633 fluorescence.

### RT-PCR assay

4T1 cells were seeded in a 6-well plate (3 × 10^5^ cells per well) and then treated with **QN-1** at various concentrations for 24 h. After that, total RNA was extracted and used as a template for reverse transcription with the following protocol (PrimeScript™ RT reagent Kit): each 20 μl reaction contained 4 μl of 5 × PrimeScript Buffer, 1 μl of 50 μM Oligo dT18 Primer, 1 μl of PrimeScript RT Enzyme Mix, DEPC-H2O, and 0.4 μg of total RNA. The mixtures were incubated at 42°C for 15 min and then at 85°C for 10 s. Afterward, PCR was performed on a PCR apparatus. The 20 μl PCR reaction mixtures contained 10 μl of 2 × HiFiTaq PCR StarMix (GenStar), 1 μl of the forward and reverse primers (10 μM), 2 μl of cDNA and nuclease-free water to volume. The program used for all genes consisted of a denaturing cycle of 5 min at 95°C and 28 cycles of PCR (95°C for 30 s, 58°C for 30 s and 72°C for 40 s). The PCR products were confirmed with agarose gel electrophoresis. The primers used in the RT-PCR are shown in the Supplementary Data.

### Western blotting

4T1 cells were incubated in a 6-well plate (3 × 10^5^ cells per well) and then treated with **QN-1** at various concentrations for 24 h. After that, cells were washed with PBS, lysed with extraction buffer using a freeze–thaw method (quickly freezing at −85°C and thawing at 4°C), and then centrifuged at 15,000 rpm at 4°C for 15 min to harvest the supernatant. The protein concentration was calculated with a BCA protein assay kit (Thermo Fisher Scientific). An equal amount of protein (30 μg) was electrophoresed on a 10% SDS−PAGE gel and transferred to a nitrocellulose membrane at 100 V for 2 h. The membrane was blocked for 1 h with a 5% nonfat dry milk solution in TBS containing 0.1% Tween-20 (TBST) at room temperature. The membrane was incubated overnight at 4°C with the primary antibody. After three washes in TBST, the membrane was incubated with the appropriate HRP-conjugated secondary antibody at room temperature for 2 h.

### Cell viability assay

Cell viability was evaluated using a Cell Counting Kit-8 (CCK8). Cells were seeded in 96-well plates (5 × 10^3^ cells per well) and exposed to various concentrations of **QN-1**. After a 24-h treatment, 10 μl of CCK8 solution was added to each well, and the cells were further incubated for 2 h. Then, the optical density (OD) was recorded at 450 nm. All experiments were performed in triplicate, and the half maximal inhibitory concentrations (IC_50_) were obtained from the curves of the mean OD values of the triplicate tests plotted against the drug concentrations. The cell lines used in this study include TNBC 4T1, lung carcinoma A549, colon carcinoma CT26WT and normal skin fibroblast BJ.

### Colony formation assay

4T1 cells were seeded in a six-well plate (300 cells per well) and exposed to **QN-1** at various concentrations at 37°C in a 5% CO_2_ incubator for 7 days (the medium with drug was replaced every three days). After that, cells were fixed with methanol and stained with crystal violet. Then, photos were taken.

### Wound scrape assay

4T1 cells were seeded in a 6-well plate (5 × 10^5^ cells per well), and cultured for 48 h. A small wound area was made in the confluent monolayer with a 200 μl pipet tip in a lengthwise stripe. Cells were then washed twice with PBS and exposed to **QN-1** at various concentrations in serum-free DMEM medium at 37°C in a 5% CO_2_ incubator for 24 h. Images were then captured.

### MTD study

A maximum-tolerated dose (MTD) study was first conducted to help determine the doses used in the *in vivo* study. The solubility of **QN-1** in DMSO is about 14 mg/ml. No more than a quarter of its LD50 (6 ml/kg, intraperitoneally) is recommended when using DMSO for toxicological investigations. Thus, in this case, the maximum drug dose we could use is determined as 21 mg/kg. We next used this dose in the MTD study. Five BALB/c mice were administrated intraperitoneally with **QN-1** (single injection), and then body weight, general activity and appearance were observed every day. We found that, during the experiment, all of the mice appeared healthy, and the weight loss was less than 15%. Therefore, this maximum dose was designated as the MTD. In the following *in vivo* experiments, the drug doses were set as 1/2, 1/4 and 1/8 of MTD (about 10, 5 and 2.5 mg/kg), respectively.

### Breast cancer mouse model experiment

Female BALB/c mice (5-week old) were acclimatized at the animal facility with pathogen-free conditions (12 h light−dark cycle at 24 ± 1°C with 60% humidity). 4T1 cells were harvested and resuspended in PBS at 2 × 10^6^ cells/ml. Each mouse was injected subcutaneously in the right flank with 2 × 10^5^ cells. Tumor growth was examined twice a week until the tumor volume reached approximately 50 mm^3^. The volume of the tumor was measured with an electronic caliper and calculated as 1/2 × length × width^2^ in mm^3^. The mice were randomly divided into five groups (7 mice each group) and treated intraperitoneally every three days for the entire observation period (20 days). Mice in the **QN-1**-treated group were administered a dose of 2.5, 5 or 10 mg/kg, those in the doxorubicin-treated group were given a dose of 2.5 mg/kg, and those in the control group were treated with saline. The tumor size and the body weight of the mice were measured every other day after drug treatment. At the end of the observation period, the mice were euthanized by cervical dislocation, and the tumors were removed and weighed. The tumor growth inhibition (TGI) was calculated according to the following formula: TGI = (1 − mean tumor weight of the experimental group/mean tumor weight of the control group) × 100%.

## RESULTS AND DISCUSSION

### Design and synthesis of quinoxaline analogs

Quinoxaline and its derivatives show very interesting biological properties, ensuring them a bright future in medicinal chemistry. Notably, diversely substituted quinoxalines are becoming recognized as a novel class of chemotherapeutic agents by targeting tubulin, topoisomerase, kinases or nucleic acids (35). Enlightened by these studies, we wanted to accommodate this skeleton the into the aryl-substituted imidazole structure (a good *c-MYC* G4 ligand but with high molecular weight and poor drug-likeness) (29), and thus designed a serial of quinoxaline analogs that possess appropriate molecular weights and potential drug-likeness. We supposed that such ligands might retain the ability to stabilize the *c-MYC* G4.

**Scheme 1.**
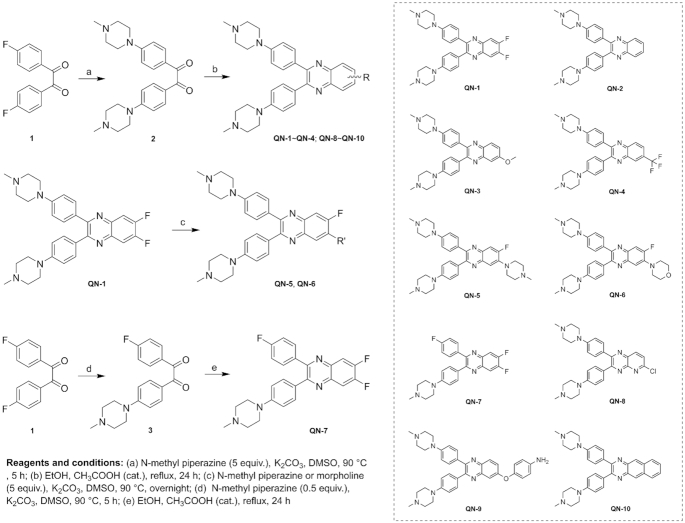
Synthesis of the quinoxaline analogs **QN-1** to **QN-10**.

Subsequently, we designed an efficient, facile synthetic route that enabled the generation of a focused library of 10 quinoxaline analogs. As shown in Scheme 1, the aryl-substituted quinoxaline scaffold was easily constructed through a condensation of an *o*-phenylenediamine and a benzyl with a high yield, described in detail in the experimental section. The structures and purities of the target compounds were confirmed by ^1^H and ^13^C NMR spectrometry, HRMS spectrometry and HPLC analysis.

### Structures of quinoxaline analogs influence *c-MYC* G4 binding and TNBC cell inhibition

The 10 compounds were evaluated for affinities to the *c-MYC* G4 and effects on cancer cell viability. Affinities were first generated by measuring the dissociation constants (*K*_D_) values between the quinoxaline analogs and the *c-MYC* G4 (pu27, the sequence is shown in Supplementary Table S1), which were determined by fitting the absorption titration data to the Benesi–Hildebrand equation (Supplementary Table S2) (34). Then, the cell proliferation inhibitory activities (IC_50_) of the quinoxaline analogs on 4T1 cells (a typical TNBC cell line) (36) were evaluated through CCK8 assays (Supplementary Table S3). From this effort, we concluded that to a great extent, the binding affinities of quinoxaline analogs to the *c-MYC* G4 were consistent with their cell growth inhibitory effects on 4T1, suggesting the quinoxaline compounds might inhibit cell proliferation mainly through targeting the *c-MYC* G4 and thus disrupting *c-MYC* transcription. The structure–activity relationship (SAR) was further explored as described below.

**Figure 1.**
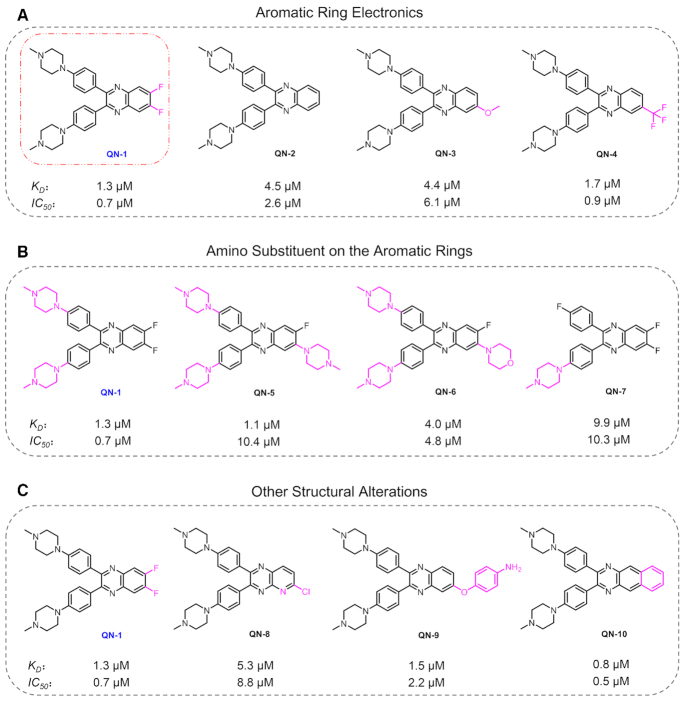
The structure–activity relationship of quinoxaline analogs. IC_50_ is the half maximum concentration for cytotoxicity in TNBC cells (4T1) at 24 h, determined by CCK8 assays; *K*_D_ is the dissociation constant between ligand and *c-MYC* G4, determined by absorption titration assays.

We first investigated the effects of electron withdrawing or donating groups on the quinoxaline skeleton. As shown in Figure 1A, introduction of electron withdrawing groups onto the quinoxaline skeleton yielded increased affinity to the *c-MYC* G4 and enhanced cell inhibitory activity (**QN-1**/**QN-4***vs***QN-2**). Conversely, electron donating substituents for electron withdrawing groups resulted in decreased activity (**QN-3** versus **QN-4**), likely indicating a weaker interaction for the aromatic ring with the electron rich guanine tetrads. Hence, the difluoro-substituted quinoxaline **QN-1** was identified as the most potent ligand.

Since amino side chains of a ligand play an important role in its recognition to G4, the number of amino substituents on the benzene rings was next evaluated. As shown in Figure 1B, the two *N*-methyl piperazine groups in **QN-1** were essential to the G4 binding, and removal of any *N*-methyl piperazine group would attenuate its G4 binding ability, thus weakening its cellular activity to a great extent (**QN-1** versus **QN-7**). On the other hand, the introduction of an extra positively charged amino substituent onto the quinoxaline skeleton somehow strengthened the interaction between ligand and *c-MYC* G4, but such a modification might influence its permeability across cell membranes, and then reduced the cytotoxicity (**QN-1** versus **QN-5**). Notably, if a neutral amino substituent was introduced, the binding affinity to *c-MYC* G4 would conversely decrease, suggesting the bulky substitution on this position was adverse for the binding (**QN-1** versus **QN-6**). Overall, two amino side chains on the quinoxaline skeleton are the best.

Some other information was obtained when analyzing the SAR (Figure 1C). First, we observed that the structural alteration of benzene to pyridine significantly attenuated its binding affinity and cytotoxicity, suggesting the quinoxaline skeleton was prerequisite (**QN-1** versus **QN-8**). Then, although the methoxy group on the quinoxaline skeleton was unfavorable for binding, introducing another aromatic substituent that might contribute to the binding (e.g. aniline) would enhance the activity (**QN-3** versus **QN-9**). It is noteworthy that fusing of one more benzene ring largely enhanced its activity (**QN-2** versus **QN-10**), probably owing to the tighter recognition between the more coplanar molecule and the *c-MYC* G4. However, this potent ligand had considerable binding affinity to double-stranded DNA (Supplementary Figure S1), which might have unexpected side effects *in vivo*.

Taken together, **QN-1**, which possesses potential drug-likeness with a suitable molecular weight (compared with most of the reported G4 ligands), displayed the best combination of G4 binding affinity and ability to inhibit TNBC cell growth. Therefore, this compound was selected for further study.

### QN-1 selectively binds and stabilizes the *c-MYC* G4

The above experiments demonstrated that **QN-1** effectively bound to the *c-MYC* G4 with a *K*_D_ of 1.3 μM (Supplementary Figure S2), which was further supported by SPR study (Supplementary Figure S3). To further understand the interactions of **QN-1** with different G4s, we again used absorption titration assays to determine their binding affinities. Interestingly, among a variety of G4s from *c-MYC* (pu27 and pu22), *BCL-2* (37), *c-KIT* (38), *VEGF* (39) and *HRAS* (40) genes and telomere (41), **QN-1** exhibited some binding preference to the *c-MYC* G4 (2.8- to 4.0-fold stronger, Supplementary Figure S2). In addition, **QN-1** did not bind to single- or double-stranded DNAs (negligible change was observed in the absorption spectra of **QN-1** titrated with DNAs, see Supplementary Figure S4). Such data indicated the potential of **QN-1** to be a selective *c-MYC* G4 ligand.

**Figure 2.**
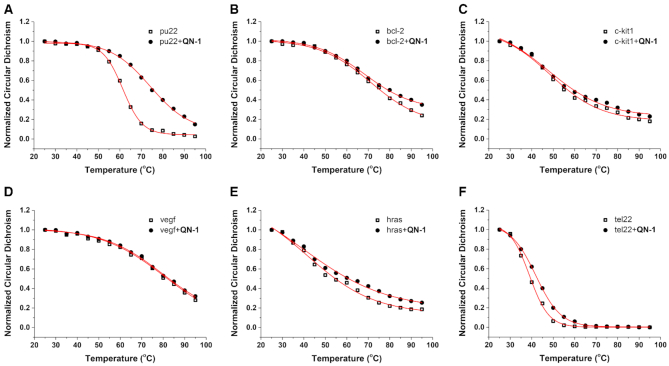
CD melting curves for different types of G4s in the absence and presence of 1 molar equivalent of **QN-1**, the concentration of KCl in Tris–HCl buffer was set at 0.5 mM: (**A**) parallel *c-MYC* G4 pu22, (**B**) parallel G4 bcl-2, (**C**) parallel G4 c-kit1, (**D**) parallel G4 vegf, (**E**) antiparallel G4 hras and (**F**) hybrid telomeric G4 tel22.

An ideal G4 ligand should possess two essential features: high G4 binding specificity and high G4 stabilizing ability. It has been proven that to some extent **QN-1** selectively targeted the *c-MYC* G4 over other G4s. To test its stabilizing ability on the *c-MYC* G4, we performed circular dichroism (CD) melting studies (Figure 2 and Supplementary Table S4). In the presence of **QN-1**, the melting temperature (*T*_m_) of pu22 increased by 13°C. In addition, the *T*_m_ of a fuller *c-MYC* sequence (pu27) was also significantly enhanced (Supplementary Figure S5), indicating this compound was highly effective at stabilizing the *c-MYC* G4. In contrast, **QN-1** had a much weaker effect on the *T*_m_ of other G4s, including hybrid G4 (tel22), antiparallel G4 (hras) and other parallel promoter G4s (bcl-2, c-kit1 and vegf), with the Δ*T*_m_ values ranging from 0 to 3°C (Supplementary Table S4). Meanwhile, **QN-1** had a negligible impact on the CD spectra of these G4s (Supplementary Figure S6). Taken together, **QN-1** preferred to bind and stabilize the *c-MYC* G4, with weaker binding to other G4s, which was distinguished from most of the reported G4 ligands.

### QN-1 binds to 5′-End G-Tetrad of the *c-MYC* G4

**Figure 3.**
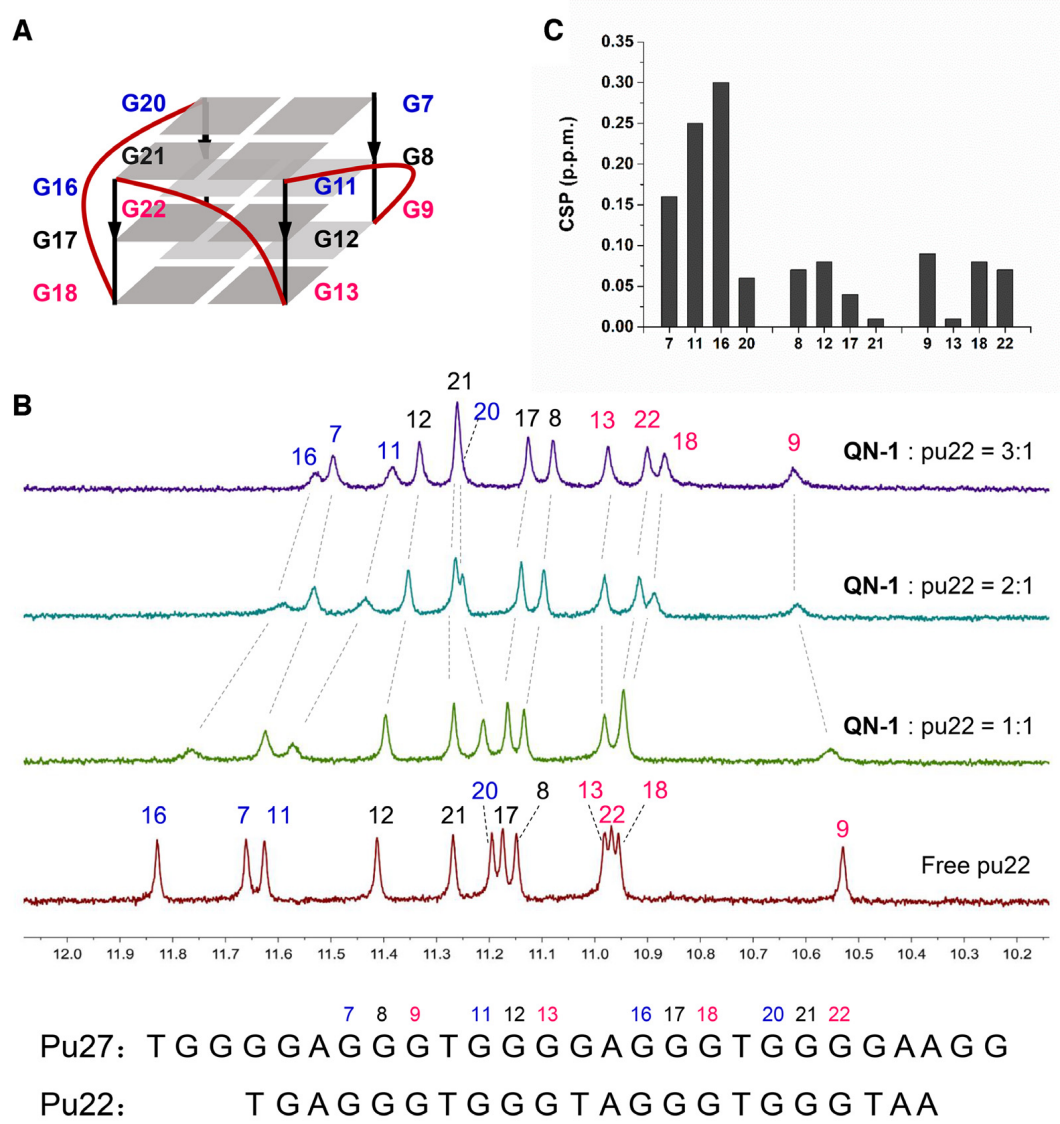
Chemical shift perturbation (CSP) analysis of *c-MYC* G4 with **QN-1**. (**A**) The folding topology of *c-MYC* G4 adopted by pu22 in K^+^ solution. The imino protons from the 5′-end G-tetrad are colored in blue, the middle G-tetrad in black, and the 3′-end G-tetrad in red. (**B**) Imino proton regions of the 1D ^1^H NMR spectra of pu22 either alone or with **QN-1**. Molar ratios of pu22: **QN-1** are as indicated at 1:0, 1:1, 1:2 and 1:3. The G-tetrad imino protons are labeled in the spectra. (**C**) CSP of the pu22 protons between the free form and the 3:1 **QN-1**–pu22 complex. The assay was performed in 25 mM KH_2_PO_4_ buffer (70 mM KCl, 10% D_2_O, pH 7.4) using 600 MHz Bruker NMR spectrometer at 25°C.

To probe the structural origin of the interaction between **QN-1** and the *c-MYC* G4, ^1^H-NMR titration was first conducted. We monitored the 1D ^1^H-NMR spectra of the *c-MYC* G4 (pu22 G14T/G23T, Figure 3A) with increasing concentrations of **QN-1**. As shown in Figure 3B, the free pu22 forms a single G4 conformation as indicated by 12 well-resolved guanine imino proton peaks, which come from the three G-tetrad planes of *c-MYC* G4 (23). Upon addition of **QN-1**, fast exchange chemical shift perturbations were observed, allowing all 12 guanine imino protons from the G-tetrad planes to be tracked. The observation of a new set of 12 well-resolved imino proton peaks suggested the formation of a well-defined **QN-1**–pu22 complex. The largest chemical shift perturbations were observed for G7, G11 and G16 (5′-end G-tetrad), while minimal perturbations were observed for the imino protons from the 3′-end and central G-tetrads (Figure 3C). Altogether, these findings suggested **QN-1** likely stacked on the 5′-end G-tetrad of *c-MYC* G4.

Modification with fluorescent 2-aminopurine (2-Ap) in different loops has been widely used to estimate the binding modes of small-molecule ligands with G4s (42). To uncover more details on the interaction, we performed titration assays using pu22 with individual 2-Ap substitution at position 6, 15 or 23, which are located at the 5′-end, the propeller loop region and the 3′-end in pu22, respectively (Supplementary Figure S7A). The results were shown in Supplementary Figure S7B. The fluorescence of ap6 and ap15 was significantly disturbed by **QN-1** compared with ap23, indicating that these bases, which are adjacent to the 5′-end G-tetrad, might be involved in the accommodation of **QN-1** in pu22. In addition, we observed no induced CD (ICD) signal in the region corresponding to the absorbance of the bound compound (Supplementary Figure S8), indicating the possibility of end stacking of **QN-1** chromophore to the *c-MYC* G4. Taken together, we proposed that the aromatic skeleton of **QN-1** might stack onto the 5′-end G-tetrad of the *c-MYC* G4, and the two outstretched *N*-methyl piperazine side chains might effectively interact with the grooves or loop bases.

### QN-1 selectively downregulates the *c-MYC* transcription by a G4-Dependent mechanism

**Figure 4.**
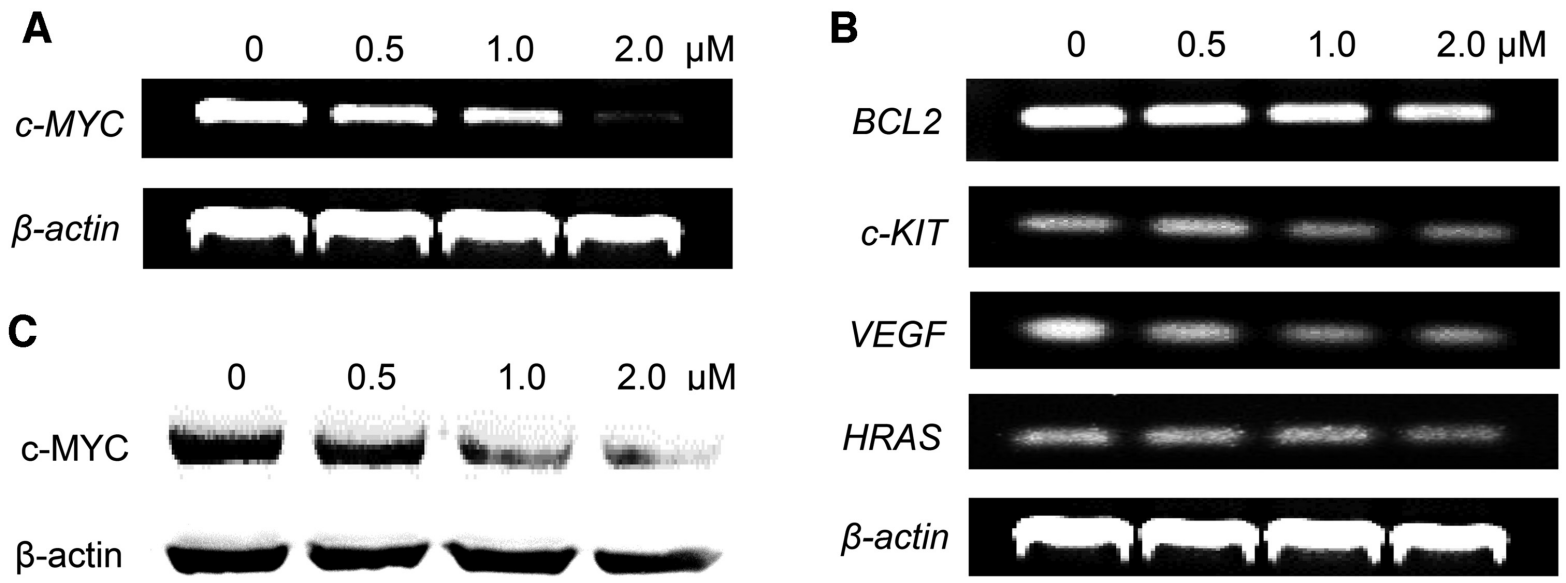
Effects of **QN-1** on the transcription of (**A**) *c-MYC* oncogene and (**B**) several other G4-driven oncogenes in 4T1 cells using RT-PCR. The reference band *β-actin* was the same one. (**C**) Effects of **QN-1** on the c-MYC expression in 4T1 cells using Western blotting.

Multiple details regarding **QN-1** targeting of the *c-MYC* G4 were evaluated *in vitro*. We next wanted to investigate whether **QN-1** selectively targets the *c-MYC* G4 and subsequently inhibits gene transcription in TNBC cells. First, RT-PCR was performed to evaluate the effect of **QN-1** on the *c-MYC* transcription in TNBC cells. 4T1 cells were treated with **QN-1** for 24 h. Then, total RNA was extracted and reverse transcribed to cDNA, which was then used as a template for PCR amplification (the primers are shown in Supplementary Table S5.). As shown in Figure 4A, 4T1 cells intrinsically had a high expression of *c-MYC* gene, and treatment of **QN-1** remarkably inhibited its transcription activity in a concentration-dependent manner. In contrast, **QN-1** had weaker effects on the transcription of a panel of G4-driven oncogenes, including *BCL-2, c-KIT, VEGF* and *HRAS* (Figure 4B), demonstrating that it had superior ability to silence *c-MYC* transcription. Next, we tested whether the subsequent c-MYC protein expression in TNBC cells was modulated by **QN-1**. Thus, 4T1 cells treated with **QN-1** for 24 h were collected for Western blotting analysis. As shown in Figure 4C, the c-MYC expression level also decreased significantly upon treatment with **QN-1**. Nevertheless, the protein expression of other G4-driven genes (*BCL-2, c-KIT, VEGF* and *HRAS*) was not significantly affected by **QN-1** (Supplementary Figure S9). The data were consistent with the trend observed in transcription modulation.

**Figure 5.**
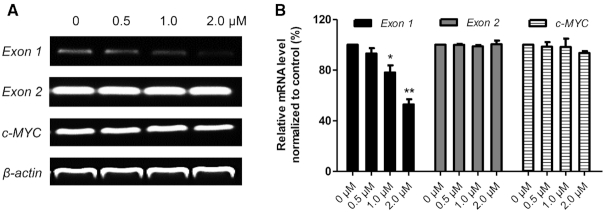
Exon-specific assays were performed in the CA46 cell line. Using primers specific to the two exons, the mRNA products of exon 1 and exon 2 in CA46 were examined independently. The experiments were repeated three times. The data are expressed as the mean ± SEM: (∗) *P* < 0.05, (∗∗) *P* < 0.01, significantly different from the control.

To assess whether the effects of **QN-1** on c-MYC in cells was G4-dependent, the CA46 exon-specific assay was performed (43–45). For most cell lines, *c-MYC* transcription is governed by the G4 located prior to exon 1 and exon 2. However, due to a translocation, in the CA46 line, only exon 1 is under G4 control (43). Thus, amplification of exon 1 represents *c-MYC* transcription from G4-maintained gene, while amplification of exon 2 represents *c-MYC* transcription from G4-lost gene. If *c-MYC* transcription is downregulated by a G4-mediated mechanism, a significant decrease in exon 1, but not exon 2, would be observed in CA46 cells (44). The results were shown in Figure 5. We observed that **QN-1** caused downregulation of transcription from exon 1, which is governed by the G4, while transcription from exon 2, which is not governed by G4, is almost unaffected. Further, as *c-MYC* transcription is not controlled by G4, mRNA level of *c-MYC* was little affected in CA46 cells treated with **QN-1**. These data suggested that the downregulation of c-MYC induced by **QN-1** was probably G4-dependent.

If a ligand induced any intracellular effects through stabilization of the *c-MYC* G4, it is expected that the cell lines with high c-MYC activation would be considerably more sensitive to the ligand. We then assessed cell viability in several cell lines treated with **QN-1**, including cancer cell lines with high expression of c-MYC (4T1 (46,47), CT26WT (48) and A549 (49), c-MYC mediates the proliferation of these cell lines) and normal BJ fibroblasts with low expression of c-MYC. As shown in Supplementary Figure S10, **QN-1** preferred to inhibit the growth of cancer cells (4T1, IC_50_ = 0.7 μM; CT26WT, IC_50_ = 0.9 μM; A549, IC_50_ = 0.8 μM) rather than normal fibroblasts (BJ, IC_50_ = 4.6 μM), suggesting that **QN-1** might remain the *c-MYC* G4 selectivity in cells. In contrast, another quinoxaline **QN-7** (Figure 1) that has weak interaction with the *c-MYC* G4 displayed much lower activity to inhibit cancer cell proliferation (4T1, IC_50_ = 10.3 μM; CT26WT, IC_50_ = 8.0 μM; A549, IC_50_ = 16.0 μM). These data demonstrated that the effects of **QN-1** on cancer cells might be largely c-MYC-dependent.

### QN-1 induced cell cycle arrest and apoptosis in TNBC cells

**Figure 6.**
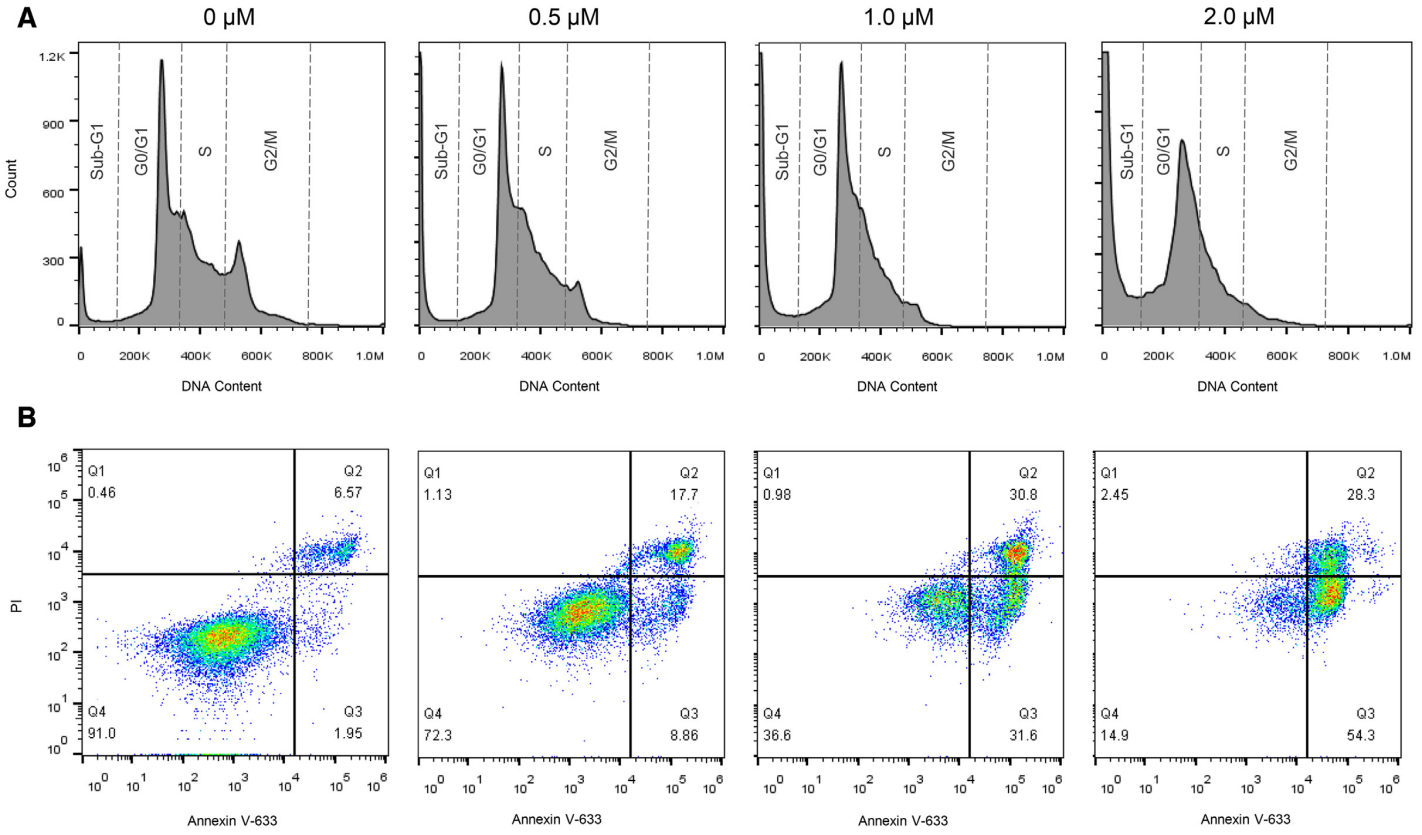
(A) Cell cycle analysis of 4T1 cells after a 24-h treatment with various concentrations of **QN-1**. The cells were collected and stained with propidium iodide (PI). The Sub-G1, G0/G1, S and G2/M phases are indicated, respectively. (B) Apoptosis evaluation of 4T1 cells after a 24-h treatment with various concentrations of **QN-1**. The cells were collected and stained with Annexin V–iFluor^TM^ 633 conjugate and PI. Region Q4 represents the viable cells; Q3 represents the early apoptotic cells; Q2 represents the late apoptotic cells.

It is sure that c-MYC regulates various cancer cellular functions, including cell cycle, apoptosis and proliferation. Basically, the effect of c-MYC on cell cycle is to drive quiescent cells into the cell cycle, thereby shortening G1 and promoting S phase. The downregulation of c-MYC should cause a preferential G1/S arrest. To determine the effect of **QN-1** on the cell cycle, 4T1 cells treated with **QN-1** at concentrations of 0, 0.5, 1.0 and 2.0 μM were analyzed using flow cytometry. As shown in Figure 6A (the statistical histogram was shown in Supplementary Figure S11), after a 24-h treatment, **QN-1** induced an apparent accumulation of cells in the G0/G1 phase dose-dependently (non-treated cells, 38.8%; **QN-1**-treated cells, 44.8%), with a concomitant loss in the G2/M phase. We also noted that the sub-G1 peak increased as the concentrations of **QN-1** increased (the percentages were 4.8%, 9.3%, 19.3% and 41.6%), indicating the robust apoptosis induced by **QN-1**. Such data were in agreement with the RT-PCR and Western blotting assays, showing the c-MYC inhibition would arrest cell cycle and induce apoptosis in TNBC cells. Moreover, we observed that the c-MYC downstream effector Cyclin D1 was significantly downregulated (Supplementary Figure S12), probably resulting from the inhibition of c-MYC expression. As Cyclin D1 is an essential regulator of the G1–S transition, whose downregulation should stall cell cycle at G1 phase, this data was consistent with the result of cell cycle analysis.

Then, we evaluated the effect of **QN-1** on the 4T1 apoptosis by Annexin V-iFluor™ 633 conjugate and PI double staining assays. As shown in Figure 6B (the statistical histogram was shown in Supplementary Figure S13), **QN-1** induced apoptotic cell death in a dose-dependent manner. In 4T1 cells treated with **QN-1** at different concentrations of 0.5, 1.0, and 2.0 μM for 24 h, the percentages of early apoptotic cells were 8.9%, 31.6% and 54.3%, and the percentages of late apoptotic cells were 17.7%, 30.8% and 28.3%, respectively. In contrast, the non-treated cells had a small population of apoptotic cells (only 2.0% for early apoptotic cells and 6.6% for late apoptotic cells). Considering the evidence, we proposed that **QN-1**, as a selective *c-MYC* G4 ligand, would block *c-MYC* transcription and then downregulate c-MYC expression, thereby arresting cell cycle at G0/G1 phase and causing cancer cell apoptosis.

### QN-1 shows promising dose-dependent anticancer activity in TNBC cells

As is reported, overexpression of c-MYC can promote the proliferation and metastasis of TNBC cells, and downregulation of c-MYC would inhibit these processes. Thus, since **QN-1** was proved to downregulate c-MYC expression, we then examined the effects of **QN-1** on the proliferation and metastasis of TNBC cells. To evaluate whether **QN-1** reduced the tumorigenicity of 4T1 cells, colony formation assays were carried out. As shown in Supplementary Figure S14A, after treatment with **QN-1** for 7 days, colony formation decreased in a dose-dependent manner. Notably, almost no colonies formed in 4T1 cells treated with 0.2 μM **QN-1**. In addition, the inhibitory effect of **QN-1** on cell migration were detected via a cell scrape assay. As shown in Supplementary Figure S14B, with an increase concentration of **QN-1**, the migration of 4T1 cells significantly decreased in the wound scrape model, suggesting its potential ability to repress TNBC metastasis. Thus, these results again proved that as a selective *c-MYC* transcription inhibitor, **QN-1** was an effective anti-TNBC molecule.

**Figure 7.**
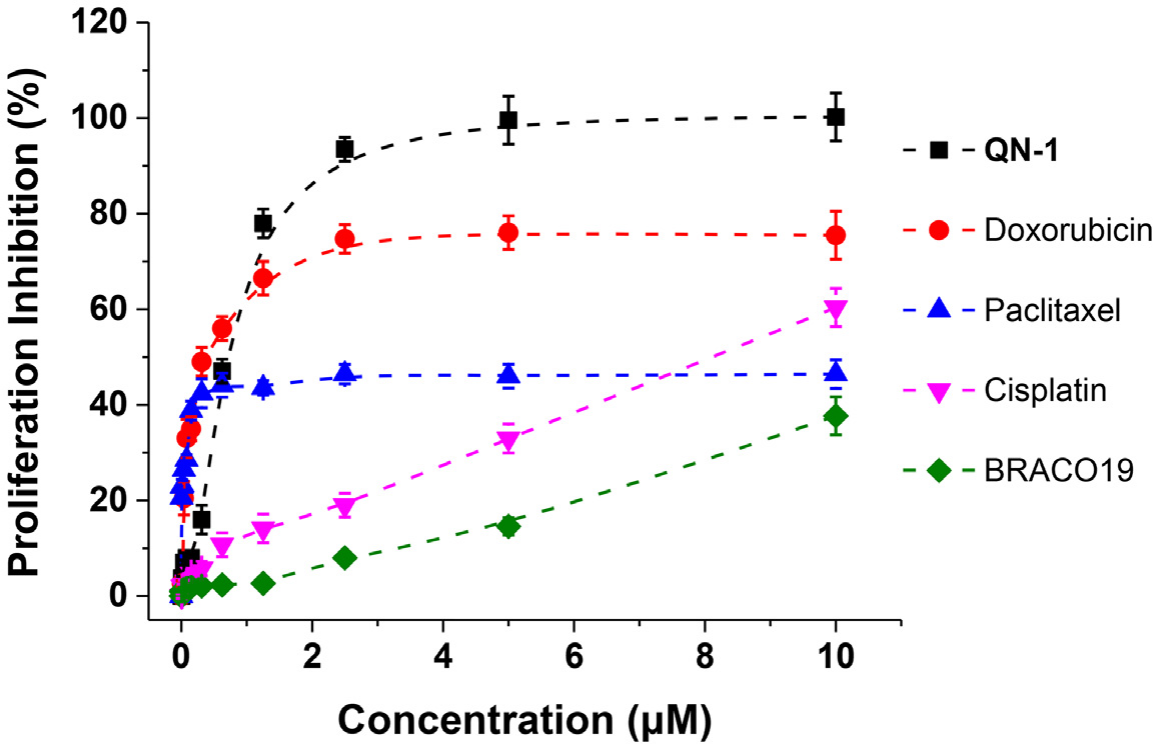
The cell growth inhibition curves of TNBC cells (4T1) after a 24-h treatment with **QN-1**, doxorubicin, paclitaxel, cisplatin and BRACO19 at different concentrations.

The above experiments demonstrated **QN-1** inhibited TNBC cell growth through being a *c-MYC* transcription down-regulator, which represented a new, alternative anti-TNBC strategy. Thus, we further compared the anticancer activity in TNBC between this compound and classical TNBC chemotherapeutics (e.g. doxorubicin, paclitaxel and cisplatin). Meanwhile, a pan G4-binding molecule BRACO19 (commercially available) was also evaluated in this assay (50). As shown in Figure 7, 4T1 cells were exquisitely sensitive to low doses of doxorubicin or paclitaxel. However, the proliferation inhibition efficiencies of these two drugs on TNBC were limited (only partial inhibition even at the concentration of 40 μM). Otherwise, **QN-1** showed a promising dose-dependent anti-TNBC activity. Of note, at the concentration of 2.5 μM, **QN-1** totally inhibited the growth of 4T1 cells. Besides, **QN-1** was much more effective on TNBC cells than BRACO19. Altogether, these findings suggested that the inhibition of *c-MYC* gene transcription by **QN-1** would become an alternative, promising anti-TNBC therapy.

### QN-1 inhibits tumor growth in a breast cancer mouse model

Next, the antitumor effect of **QN-1** was confirmed in a 4T1 tumor‐bearing mouse model, which is a suitable experimental animal model for human TNBC (36). Before this experiment, MTD was first determined as 21 mg/kg (see Materials and Methods and Supplementary Figure S15). Thus, in the following *in vivo* experiment, the drug doses were set as 1/2, 1/4 and 1/8 of MTD (about 10, 5 and 2.5 mg/kg), respectively.

**Figure 8.**
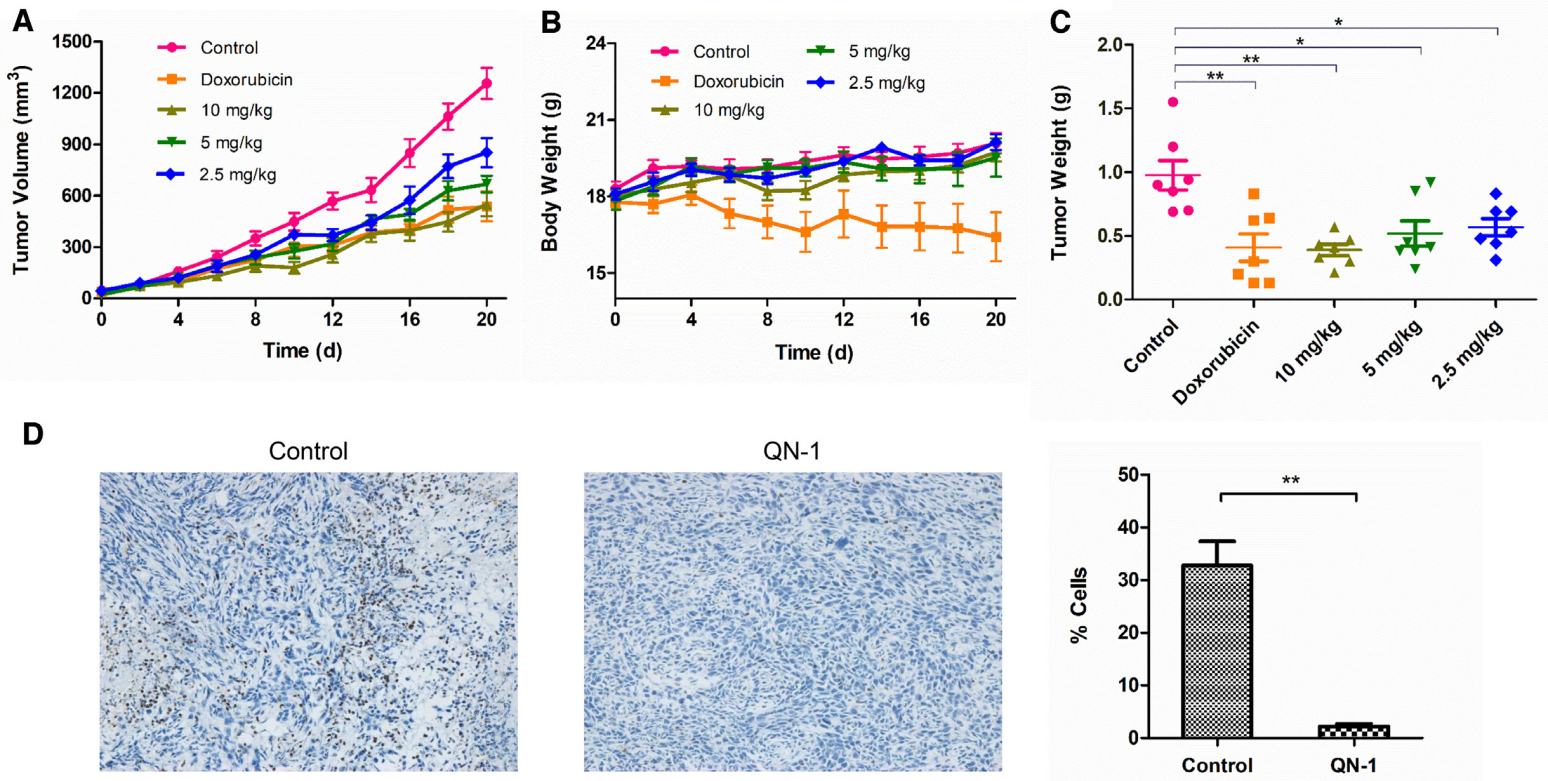
**QN-1** inhibits tumor growth in a 4T1 TNBC mouse model. After treatment with **QN-1** at 10 mg/kg, 5 mg/kg or 2.5 mg/kg or with doxorubicin at 2.5 mg/kg for 20 days, the mice were sacrificed, and the tumors were weighed. (**A**) Tumor volumes of the mice in each group during the treatment period. (**B**) Body weights of the mice in each group during the treatment period. (**C**) Weights of the excised tumors from each group when the treatment ended. (**D**) The expression of c-MYC protein (brown area) in the tumor tissues from the **QN-1**-treated groups (10 mg/kg), as determined by IHC. The percentages of c-MYC-expressing cells were analyzed. The data are presented as the mean ± SEM: (∗) *P* < 0.05, (∗∗) *P* < 0.01, significantly different from the control based on Student's t test.

The tumor-bearing BALB/c mice were divided into five groups (7 mice in each group) and treated with saline (negative control), doxorubicin (positive control, 2.5 mg/kg) and **QN-1** (three experimental groups, at doses of 10, 5 and 2.5 mg/kg) by intraperitoneal injection every three days. Tumor volume and body weight of each mouse were recorded every other day. As shown in Figure 8A, during the experiment period, **QN-1** displayed dose-dependent inhibition of tumor growth. At the dose of 10 mg/kg, **QN-1** significantly inhibited the tumor growth, which was comparable to doxorubicin. Meanwhile, body weight of the **QN-1**-treated groups remained stable throughout the study time, similar to the saline control group, indicating that **QN-1** was tolerated well at these doses (Figure 8B). However, there was a significant weight loss after treatment with doxorubicin. Besides, the **QN-1**-treated mice appeared active as the normal mice, with no signs of skin tearing, ulceration, ill health, distress or discomfort. In contrast, the doxorubicin-treated mice seemed inactive and became very weak at the end of the experiment. Overall, **QN-1** exhibited good *in vivo* anti-TNBC activity with fewer side effects.

At the end of the experiment, the mice were sacrificed, and the tumors were removed and weighed. As shown in Figure 8C, compared with the saline control group, treatment with **QN-1** at 10, 5 and 2.5 mg/kg resulted in a significant reduction in tumor weight, with tumor growth inhibition (TGI) of 60%, 47% and 42%, respectively. We also anatomized the mice and examined their viscera. We observed no obvious change in any adult organs, and found that there was no significant difference in organ weight between the saline control group and the **QN-1**-treated groups, but the organ weight of the doxorubicin-treated groups significantly decreased (Supplementary Figure S16). These data again demonstrating the good tolerance of **QN-1**.

To determine whether the expression of c-MYC was consistently affected within tumors during treatment with **QN-1**, we assessed the c-MYC expression (brown areas in Figure 8D) in tumor tissues using immunohistochemistry (IHC). The **QN-1**-treated group exhibited significantly decreased c-MYC expression compared with the saline control group. The results indicated that **QN-1** might inhibit tumor growth by specifically downregulating c-MYC expression.

## CONCLUSION

TNBC is an aggressive form of breast cancer, and the current drugs for TNBC are limited, calling for the discovery of new therapeutic drugs. Since *c-MYC* overexpression is associated with the poor outcomes in TNBC, inhibiting the *c-MYC* transcription might be a new anti-TNBC strategy. In this study, we designed and synthesized a serial of quinoxaline analogs targeting the *c-MYC* promoter G4 structure, aiming to downregulate the c-MYC expression. Next, these compounds were screened for their binding affinity to *c-MYC* G4 and cytotoxicity to TNBC cells. Among them, **QN-1** was identified as the most promising ligand. Further absorption titrations and CD melting studies revealed that, among various G4s, **QN-1** preferred to bind and stabilize the *c-MYC* G4. Meanwhile, NMR titrations and 2-Ap experiments together indicated **QN-1** staked onto the 5′-end G-tetrad of the *c-MYC* G4. Subsequently, RT-PCR, Western blotting and CA46 exon-specific assay revealed that **QN-1** selectively downregulated *c-MYC* transcription and expression in TNBC cells by specifically targeting *c-MYC* G4. We hence evaluated the effects of **QN-1** on TNBC cells, demonstrating that it could provoke cell cycle arrest and apoptosis, repress metastasis and inhibit cancer cell growth, which might be ascribed to downregulation of c-MYC expression. Furthermore, **QN-1** also exhibited good *in vivo* antitumor ability in 4T1 tumor-bearing mice. Compared with the previously reported *c-MYC* G4 ligand **IZCZ-3** (29), **QN-1** retains the good selectivity to the *c-MYC* G4 accompanied with several advantages. First, the core structure is more drug-like, and represents a new, promising scaffold for targeting *c-MYC* G4. Then, **QN-1** appears much more potent to inhibit tumor growth, showing effective inhibition for TNBC. Besides, such a structure is easier to be synthesized. This work provides new insights for the development of alternative anti-TNBC drugs that specifically target the *c-MYC* G4 and thus inhibit the c*-MYC* transcription.

## Supplementary Material

gkz835_Supplemental_File
