# Supplementary material for: New substituted quinoxalines inhibit triple-negative breast cancer by specifically downregulating the c-MYC transcription
Source: Nucleic Acids Res. 2019 Oct 4;47(20):10529–42. doi: 10.1093/nar/gkz835 (PMC6846596; doi:10.1093/nar/gkz835)
Supplement: gkz835_Supplemental_File [file gkz835_supplemental_file.docx]

**SUPPLEMENTARY DATA**

**New Substituted Quinoxalines Inhibit Triple-Negative Breast Cancer by Specifically Downregulating the *c-MYC* Transcription**

Ming-Hao Hu,* Tian-Ying Wu, Qiong Huang, Guangyi Jin*

**Table of Contents:**

| **Table S1.** The oligonucleotides used in the present study | S2 |
| --- | --- |
| **Table S2.** Dissociation constants between quinoxaline analogs and *c-MYC* G4 | S2 |
| **Table S3.** IC_50_ values of quinoxaline analogs against TNBC 4T1 cells | S2 |
| **Figure S1.** UV-vis absorption titrations of **QN-10** with duplex DNA | S3 |
| **Figure S2.** Dissociation constants between **QN-1** and various DNAs | S3 |
| **Figure S3.** SPR curves of **QN-1** interacting with the *c-MYC* G4 | S4 |
| **Figure S4.** UV-vis absorption titrations of **QN-1** with single- or double-stranded DNAs | S4 |
| **Table S4.** The G4-stabilizing ability of **QN-1** determined by CD melting studies | S4 |
| **Figure S5.** CD melting curves for pu27 in the absence and presence of **QN-1** | S5 |
| **Figure S6.** CD spectra for the G4s with and without **QN-1** in Tris-HCl buffer | S5 |
| **Figure S7.** Plot of fluorescence of 2-Ap labelled pu22 versus binding ratio of [**QN-1**]/[DNA] | S6 |
| **Figure S8.** CD spectra of **QN-1** with or without *c-MYC* G4 pu22 | S6 |
| **Table S5.** Primer sequence of each gene used in the present study | S7 |
| **Figure S9.** Effects of **QN-1** on the protein expression of other G4-driven genes | S7 |
| **Figure S10.** Cell growth inhibition curves of 4T1, CT26WT and BJ cells treated with **QN-1** | S7 |
| **Figure S11.** Cell cycle analysis of 4T1 cells after treatment with **QN-1** | S8 |
| **Figure S12.** Effects of **QN-1** on the Cyclin D1 expression in 4T1 cells | S8 |
| **Figure S13.** Apoptosis evaluation of 4T1 cells after treatment with **QN-1** | S8 |
| **Figure S14.** Effects of **QN-1** on the colony formation and migration of 4T1 cells | S9 |
| **Figure S15.** Body weight change of BALB/c mice after injected with **QN-1** | S9 |
| **Figure S16.** Viscera weights of the mice in each group when the treatment ended | S10 |
| **Figure S17–46.** ^1^H NMR, ^13^C NMR and HRMS spectra of the final compounds | S11 |

**Table S1.** The oligonucleotides used in the present study

| Name | Sequence (5′→3′) | Structure in K^+^ Solution |
| --- | --- | --- |
| pu27 | TGGGGAGGGTGGGGAGGGTGGGGAAGG | Parallel G4 |
| pu22 | TGAGGGTGGGTAGGGTGGGTAA | Parallel G4 |
| vegf | GGGGCGGGCCGGGGGCGGGG | Parallel G4 |
| bcl-2 | GGGCGGGCGCGGGAGGAAGGGGGCGGG | Parallel G4 |
| c-kit1 | AGGGAGGGCGCTGGGAGGAGGG | Parallel G4 |
| hras | TCGGGTTGCGGGCGCAGGGCACGGGCG | Antiparallel G4 |
| tel22 | AGGGTTAGGGTTAGGGTTAGGG | Hybrid G4 |
| hairpin | CGCGCGCGTTTTCGCGCGCG | Double-Stranded DNA |
| T21 | TTTTTTTTTTTTTTTTTTTTT | Single-Stranded DNA |
| pu22-ap6 | TG(2-Ap)GGGTGGGTAGGGTGGGTAA | Parallel G4 |
| pu22-ap15 | TGAGGGTGGGT(2-Ap)GGGTGGGTAA | Parallel G4 |
| pu22-ap23 | TGAGGGTGGGTAGGGTGGG(2-Ap)AA | Parallel G4 |

**Table S2.** Dissociation constants (*K*_D_) between quinoxaline analogs and *c-MYC* G4 (pu27)

| Compound | **QN-1** | **QN-2** | **QN-3** | **QN-4** | **QN-5** |
| --- | --- | --- | --- | --- | --- |
| *K*_D_ | 1.3 μM | 4.5 μM | 4.4 μM | 1.7 μM | 1.1 μM |
| Compound | **QN-6** | **QN-7** | **QN-8** | **QN-9** | **QN-10** |
| *K*_D_ | 4.0 μM | 9.9 μM | 5.3 μM | 1.5 μM | 0.8 μM |

**Table S3.** IC_50_ values of quinoxaline analogs against TNBC 4T1 cells as determined by CCK8 assays

| Compound | **QN-1** | **QN-2** | **QN-3** | **QN-4** | **QN-5** |
| --- | --- | --- | --- | --- | --- |
| IC_50_ | 0.7 μM | 2.6 μM | 6.1 μM | 0.9 μM | 10.4 μM |
| Compound | **QN-6** | **QN-7** | **QN-8** | **QN-9** | **QN-10** |
| IC_50_ | 4.8 μM | 10.3 μM | 8.8 μM | 2.2 μM | 0.5 μM |

**
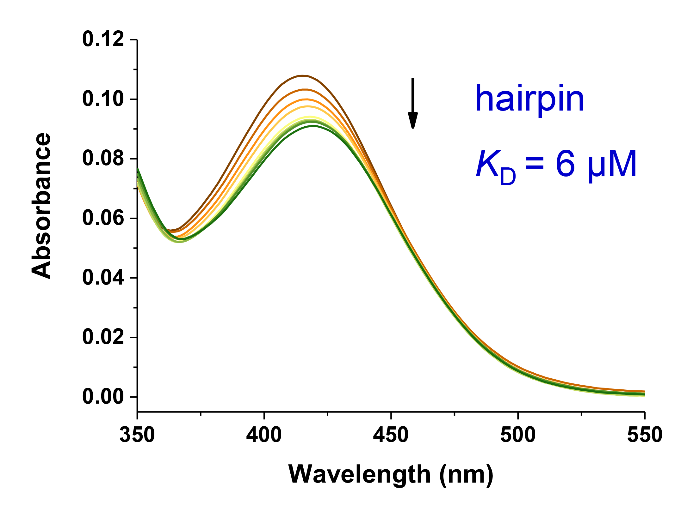
**

**Figure S1.** UV-vis absorption titration of 10 μM **QN-10** with the stepwise addition of duplex DNA (0–10 μM), and dissociation constants (*K*_D_) between **QN-10** and hairpin was determined as 6 μM.


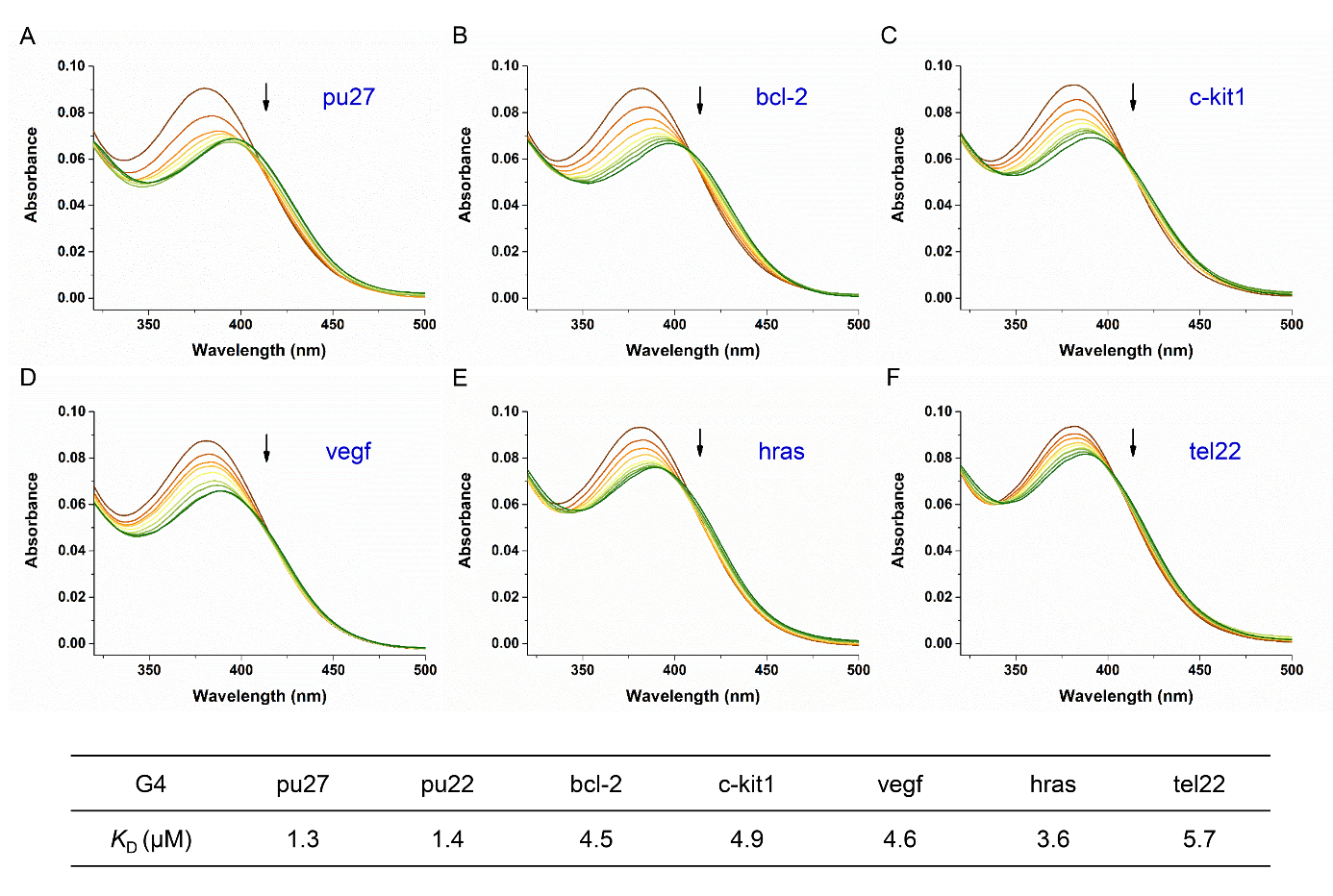


**Figure S2.** Above: UV-vis absorption titrations of 10 μM **QN-1** with the stepwise addition of G4s, including (A) pu27, (B) bcl-2, (C) c-kit1, (D) vegf, (E) hras and (F) tel22 (0–10 μM) in 10 mM Tris-HCl buffer, 100 mM KCl, pH 7.2. Arrows indicate the absorbance changes. Below: dissociation constants (*K*_D_) between **QN-1** and G4s (pu22 was included) that were determined by fitting the data to the Benesi–Hildebrand equation.


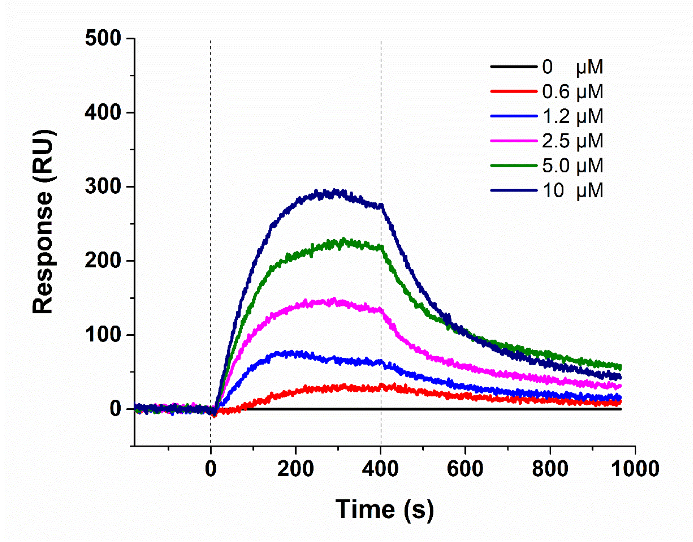


**Figure S3.** SPR curves of **QN-1** interacting with the *c-MYC* G4 (pu27). The *K*_D_ was determined as 1.5 μM.


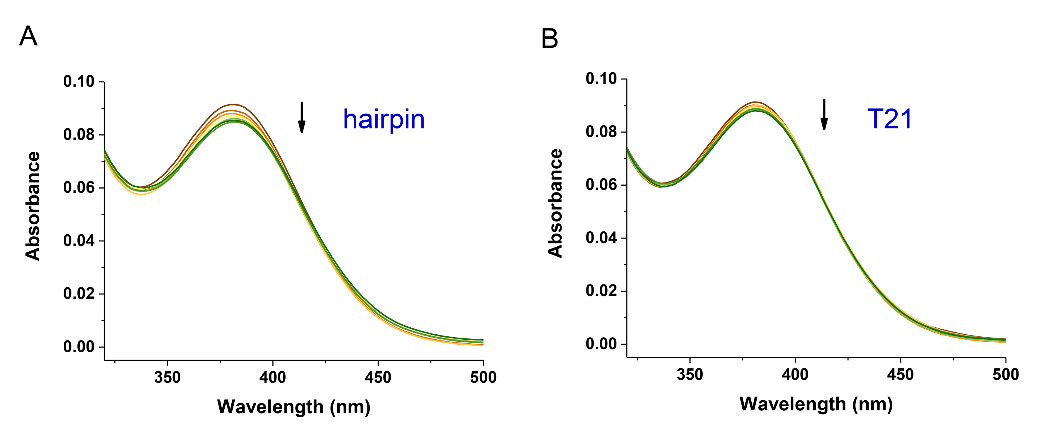


**Figure S4.** UV-vis absorption titrations of **QN-1** (10 μM) with single- or double-stranded DNAs (0–10 μM)

**Table S4.** The G4-stabilizing ability of **QN-1** ^a^

| G4 | *T*_m_ (^o^C) | | Δ*T*_m_ (^o^C) |
| --- | --- | --- | --- |
|  | Without **QN-1** | With **QN-1** |  |
| pu22 | 61 | 74 | 13 |
| bcl-2 | 71 | 73 | 2 |
| c-kit1 | 48 | 49 | 1 |
| vegf | 81 | 81 | 0 |
| hras | 40 | 42 | 2 |
| tel22 | 39 | 42 | 3 |
| ^a^ Melting temperature (*T*_m_) of G4s with and without **QN-1** detected by circular dichroism spectroscopy. | | | |


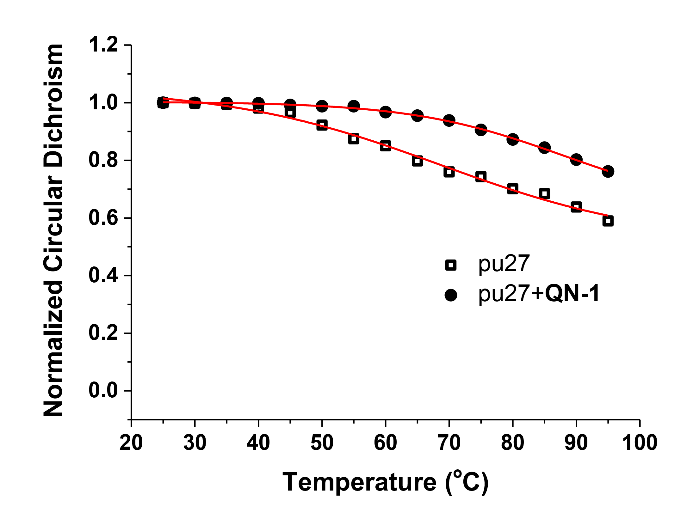


**Figure S5.** CD melting curves for the fuller *c-MYC* G4 (pu27) in the absence and presence of 1 molar equivalent of **QN-1**. We observed a significant increase in the stability of pu27 treated with **QN-1**, but the corresponding Δ*T*_m_ could not be obtained.


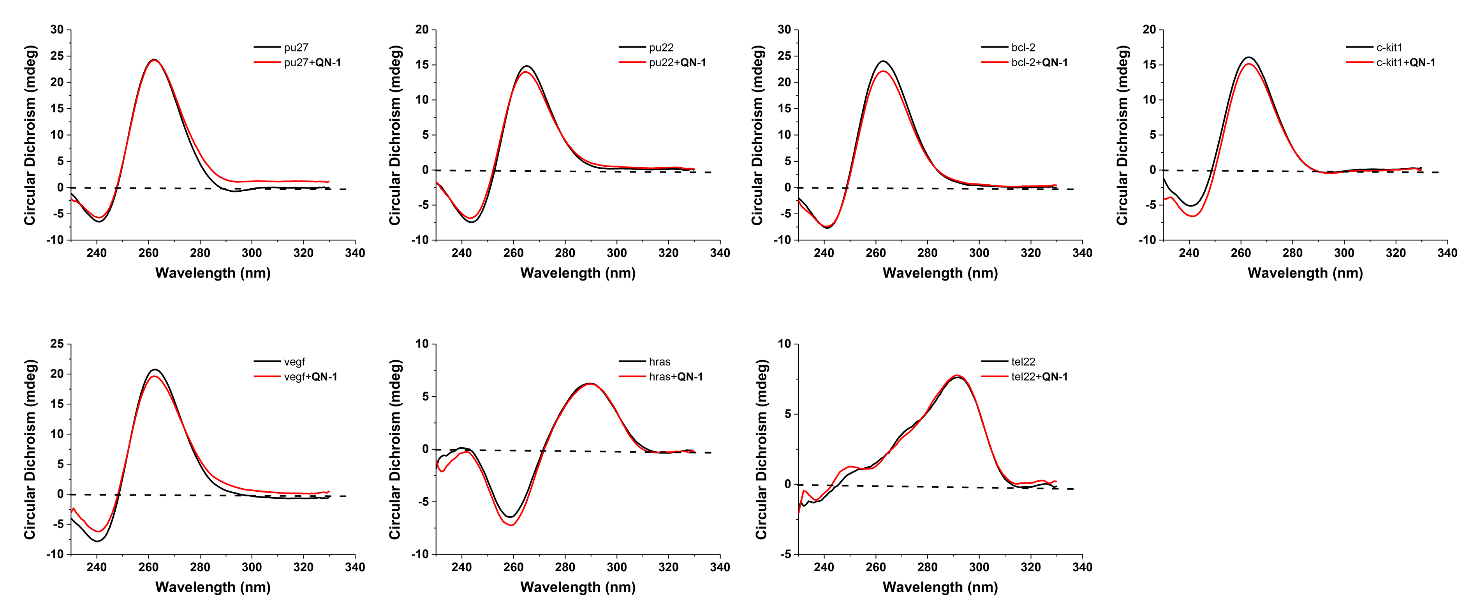


**Figure S6.** CD spectra for the G4s (including pu27, pu22, bcl-2, c-kit1, vegf, hras and tel22) with and without **QN-1** in Tris-HCl buffer. The concentrations of G4s and **QN-1** were set at 2 μM. **QN-1** had a negligible impact on the CD spectra of these G4s.


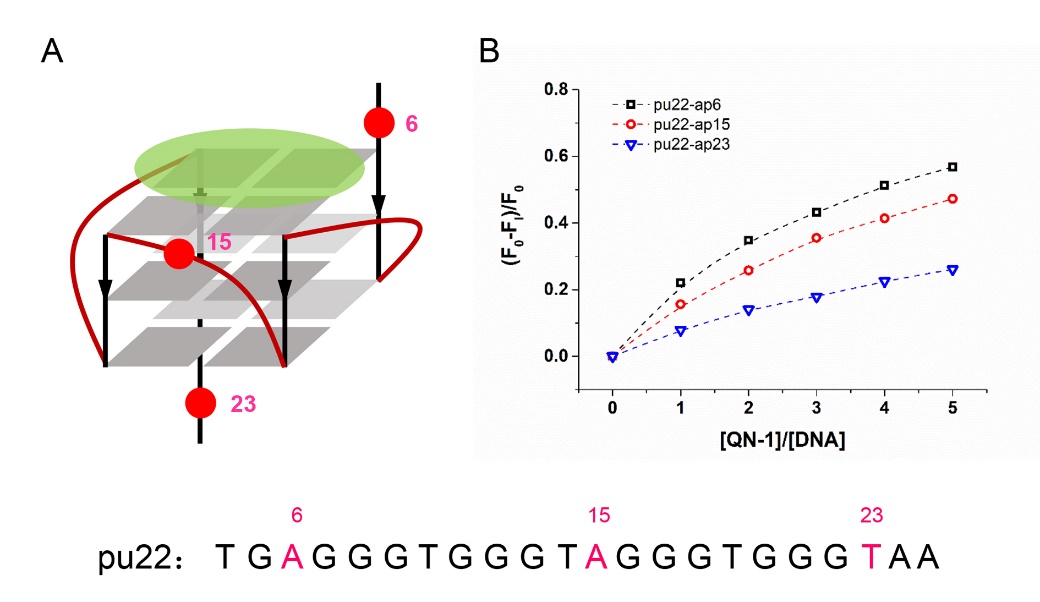


**Figure S7.** Plot of normalized fluorescence intensity at 375 nm of 2-Ap individually labelled pu22 versus binding ratio of [**QN-1**]/[DNA], λ_ex_ = 305 nm. Structure model of pu22 is provided. The positions of 2-Ap bases are marked.





**Figure S8.** CD spectra of **QN-1** (30 μM) with or without *c-MYC* G4 pu22 (30 μM) in 10 mM Tris-HCl buffer, 100 mM KCl, pH 7.2. It show no induced CD signal for **QN-1**, suggesting end-stacking of **QN-1** to pu22.

**Table S5.** Primer sequence of each gene used in the present study

| Gene | Forward primer (5′→3′) | Reverse primer (5′→3′) |
| --- | --- | --- |
| *c-MYC* | GTGGCACCTCTTGAGGACCT | TGGTGCTCCATGAGGAGACA |
| *c-KIT* | CGTGGAAAAGAGAAAACAGTCA | CACCGTGATGCCAGCTATTA |
| *VEGF* | GCACATAGAGAGAATGAGCTTCC | CTCCGCTCTGAACAAGGCT |
| *BCL-2* | GAGGATTGTGGCCTTCTTTG | GCCGGTTCAGGTACTCAGTC |
| *HRAS* | TTTGTGGACGAGTATGATCCCA | TGCTCCCTGTACTGATGGATG |
| *Exon 1* | GCTCCCTCTGCCTCTCGCTG | CGTCCCTGGCTCCCCTCCT |
| *Exon 2* | CCAGCGAGGATATCTGGAAGAA | CCGAAGGGAGAAGGGTGTGA |
| *β-actin* | GCATCCTGTCGGCAATGC | GTTGCTATCCAGGCTGTGC |


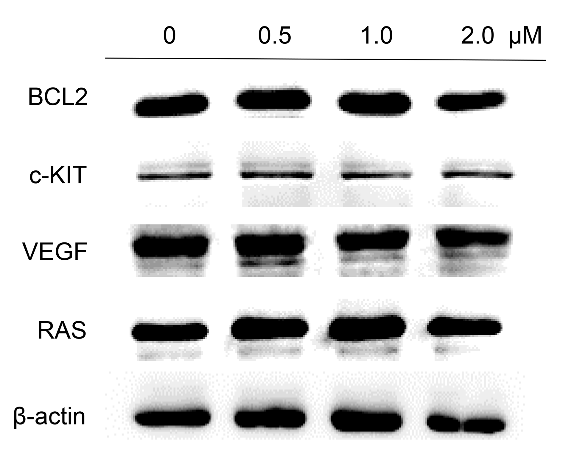


**Figure S9.** Effects of **QN-1** on the protein expression of other G4-driven genes using Western blotting





**Figure S10.** The cell growth inhibition curves of 4T1 cells, CT26WT cells, A549 cells and BJ fibroblasts after a 24-h treatment with **QN-1**. The respective IC_50_ value is 0.7 μM, 0.9 μM, 0.8 μM and 4.6 μM.





**Figure S11.** Cell cycle analysis of 4T1 cells after treatment with various concentrations of **QN-1**.


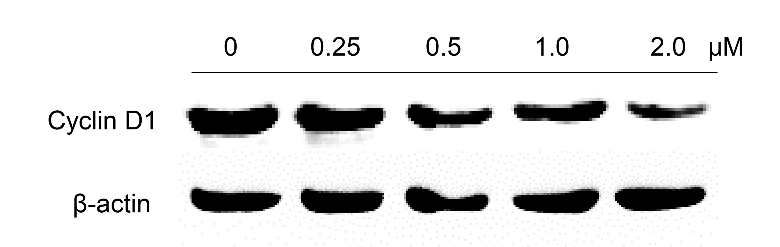


**Figure S12.** Effects of **QN-1** on the Cyclin D1 expression in 4T1 cells using Western blotting.





**Figure S13.** Apoptosis evaluation of 4T1 cells after treatment with various concentrations of **QN-1**.


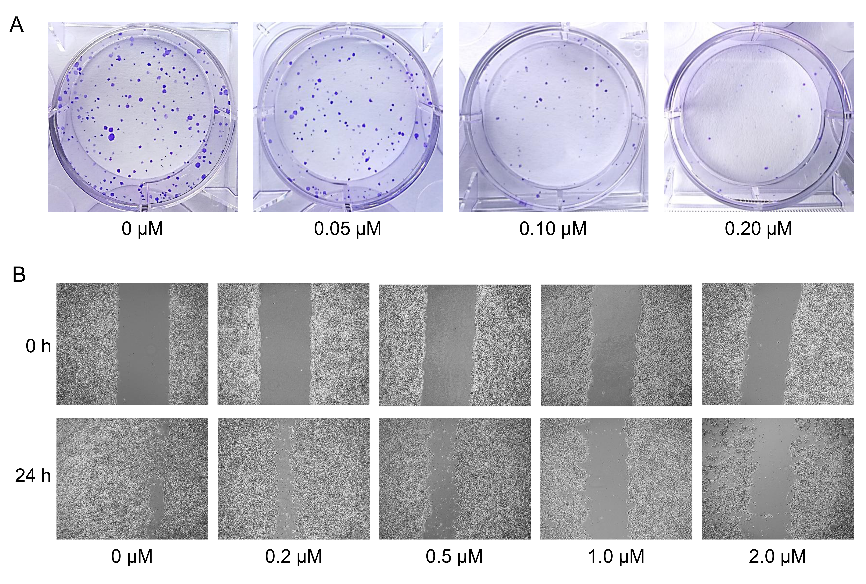


**Figure S14.** (A) Effects of **QN-1** on the colony formation of 4T1 cells. Cells were planted in a six-well dish with increasing concentrations of **QN-1** for 7 days and then stained with crystal violet. (B) Effects of **QN-1** on the migration of 4T1 cells. A cross-shaped scrape was made after the cells were confluent as a monolayer, and then the cells were treated with different concentrations of **QN-1** for 24 h in the absence of fetal bovine serum.





**Figure S15.** Body weight change of BALB/c mice after injected without or with **QN-1** at 21 mg/kg. The solubility of **QN-1** in DMSO is about 14 mg/mL. Besides, no more than a quarter of its LD50 (6 mL/kg, intraperitoneally) is recommended when using DMSO for toxicological investigations. Thus, in this case, the maximum drug dose we could use is determined as 21 mg/kg. Five BALB/c mice were administrated with **QN-1** intraperitoneally (single injection), and then body weight was observed every day.





**Figure S16.** Viscera weights of the mice in each group when the treatment ended. The data are presented as the mean ± SEM: (∗) *P* < 0.05, (∗∗) *P* < 0.01, and (∗∗∗) *P* < 0.001, significantly different from the control.

**^1^H NMR, ^13^C NMR and HRMS Spectra of the Final Compounds**


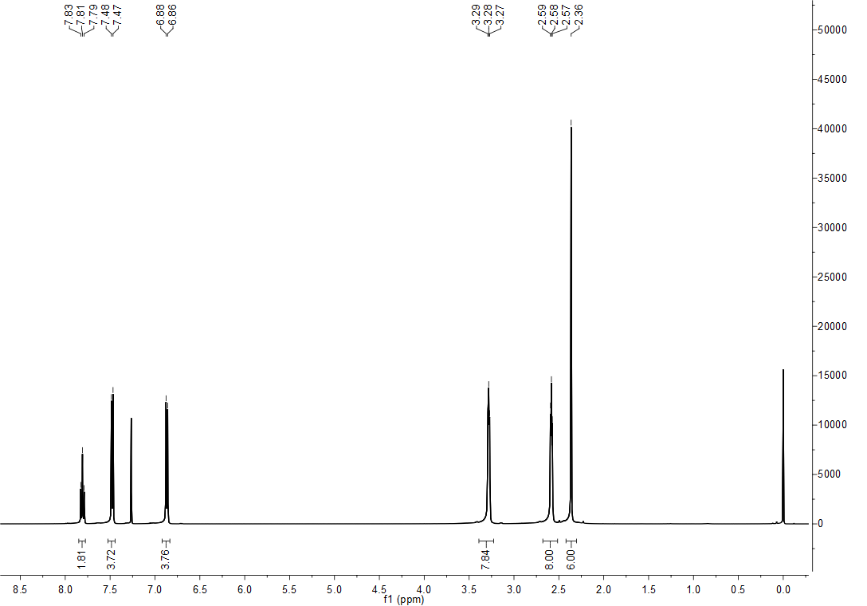


**Figure S17.** ^1^H NMR spectrum of **QN-1**


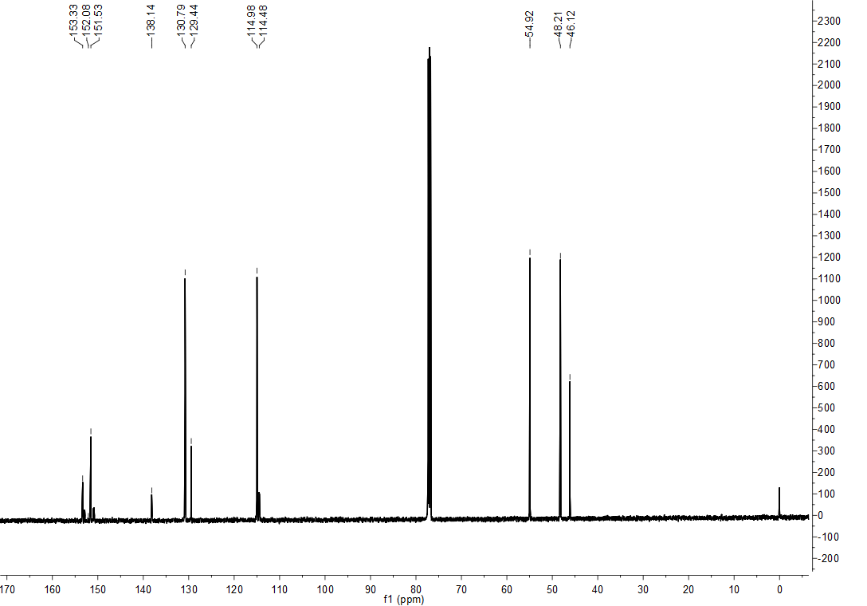


**Figure S18.** ^13^C NMR spectrum of **QN-1**

**Figure S19.** HRMS spectrum of **QN-1**


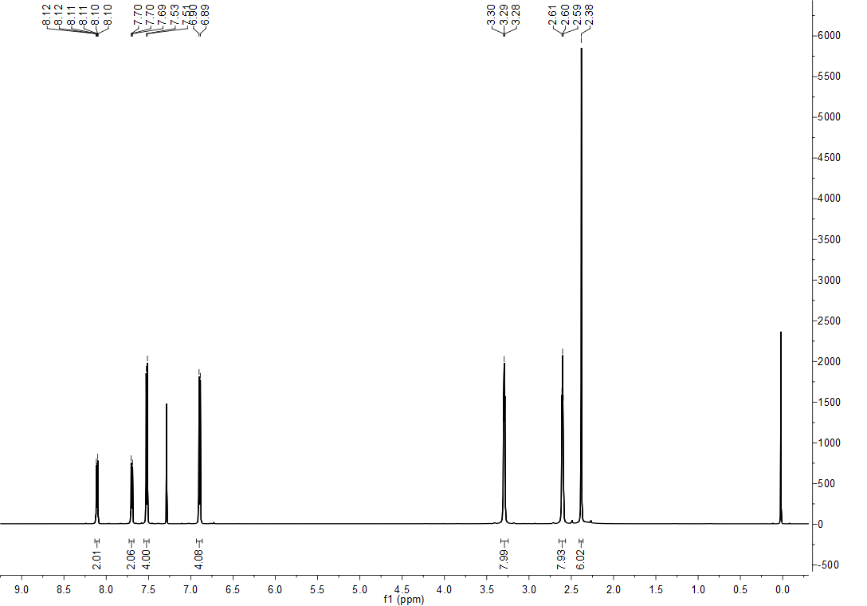


**Figure S20.** ^1^H NMR spectrum of **QN-2**


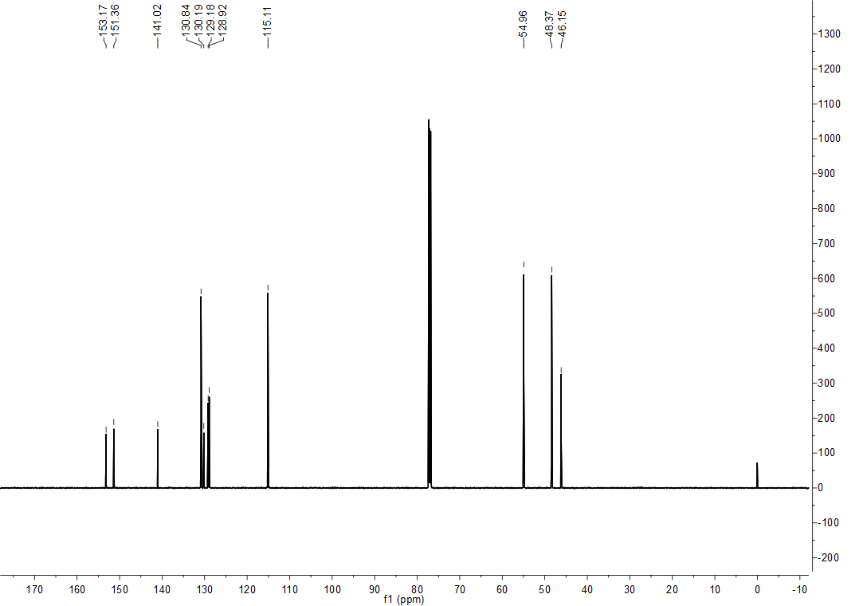


**Figure S21.** ^13^C NMR spectrum of **QN-2**

**Figure S22.** HRMS spectrum of **QN-2**


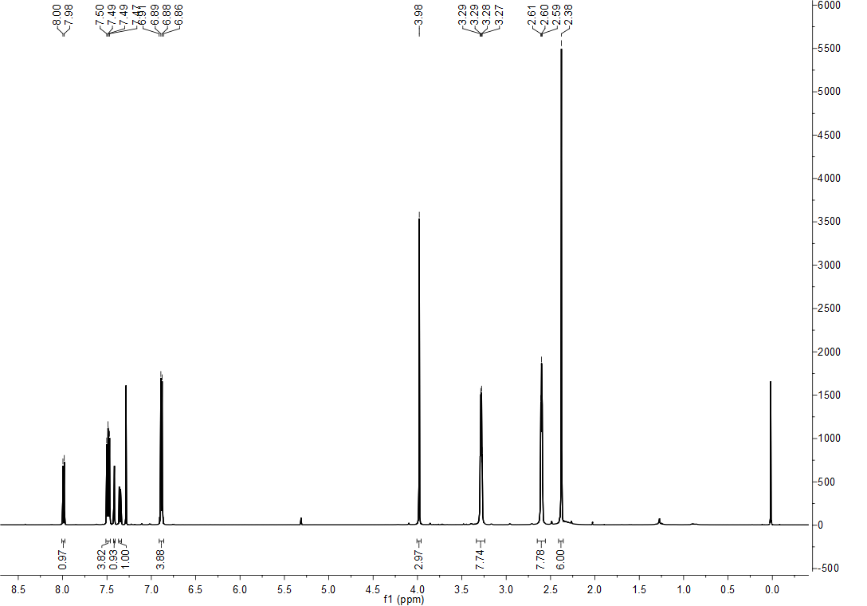


**Figure S23.** ^1^H NMR spectrum of **QN-3**


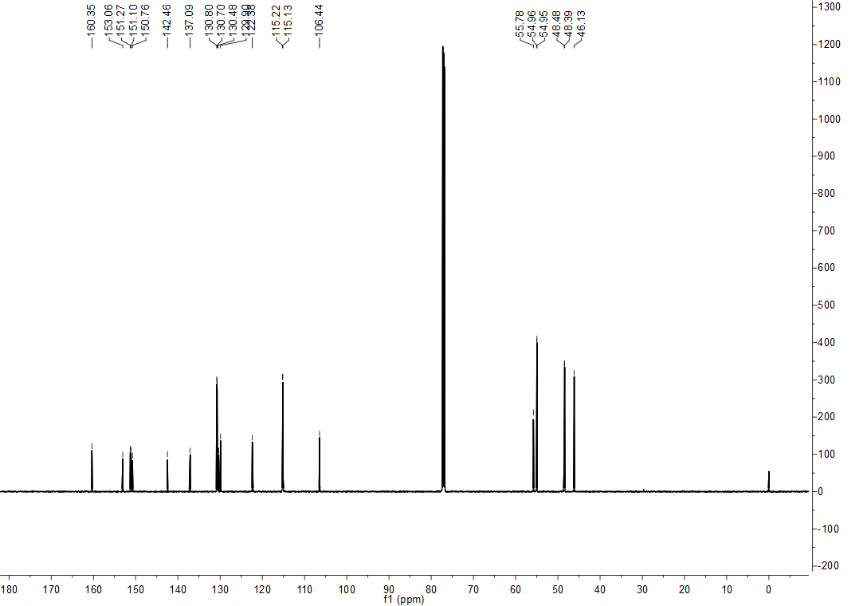


**Figure S24.** ^13^C NMR spectrum of **QN-3**

**Figure S25.** HRMS spectrum of **QN-3**


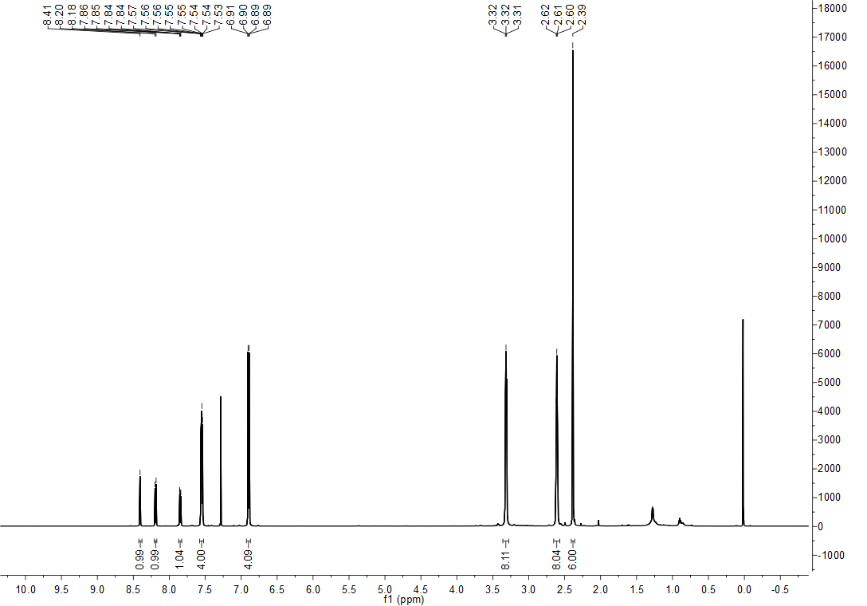


**Figure S26.** ^1^H NMR spectrum of **QN-4**


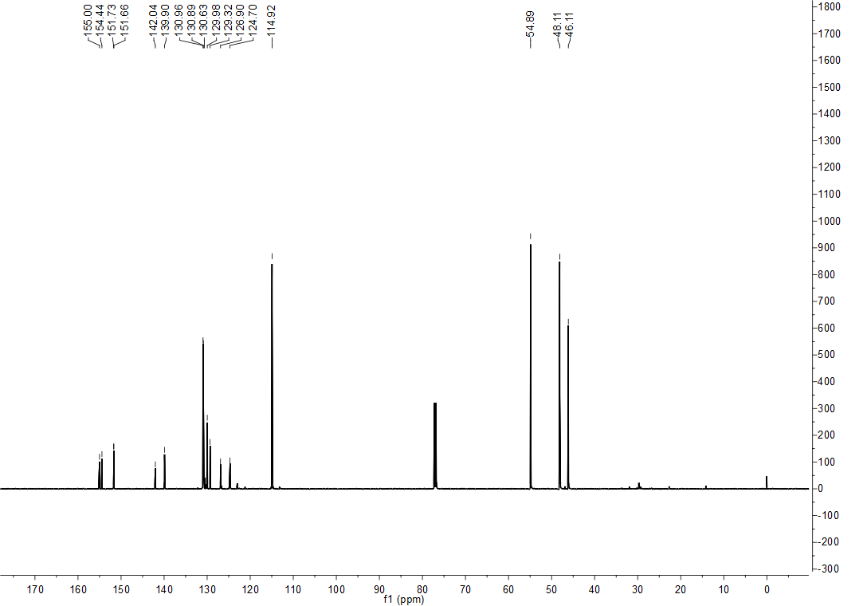


**Figure S27.** ^13^C NMR spectrum of **QN-4**

**Figure S28.** HRMS spectrum of **QN-4**


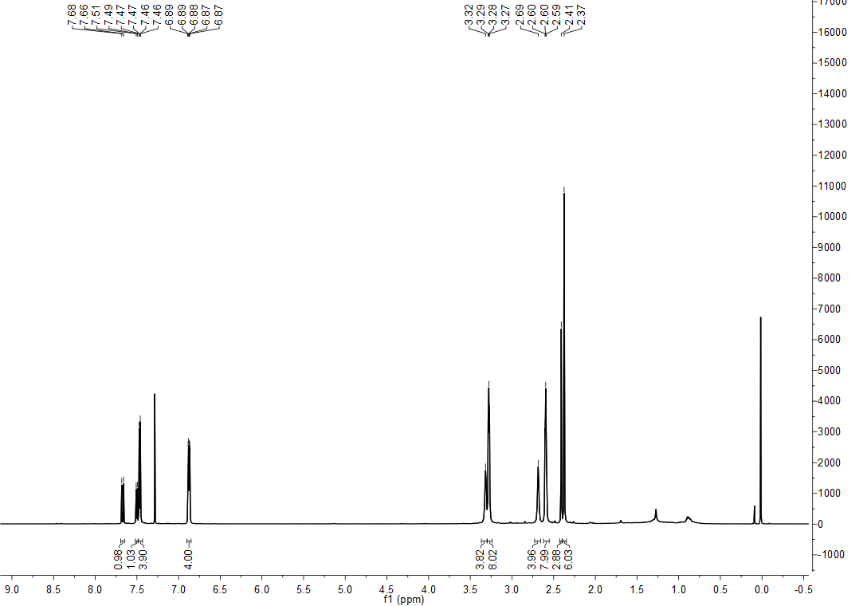


**Figure S29.** ^1^H NMR spectrum of **QN-5**


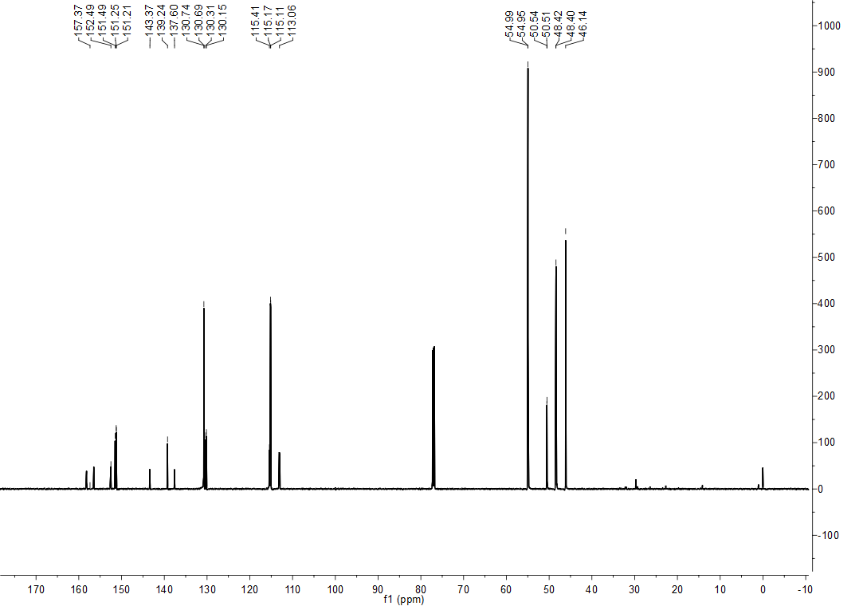


**Figure S30.** ^13^C NMR spectrum of **QN-5**

**Figure S31.** HRMS spectrum of **QN-5**


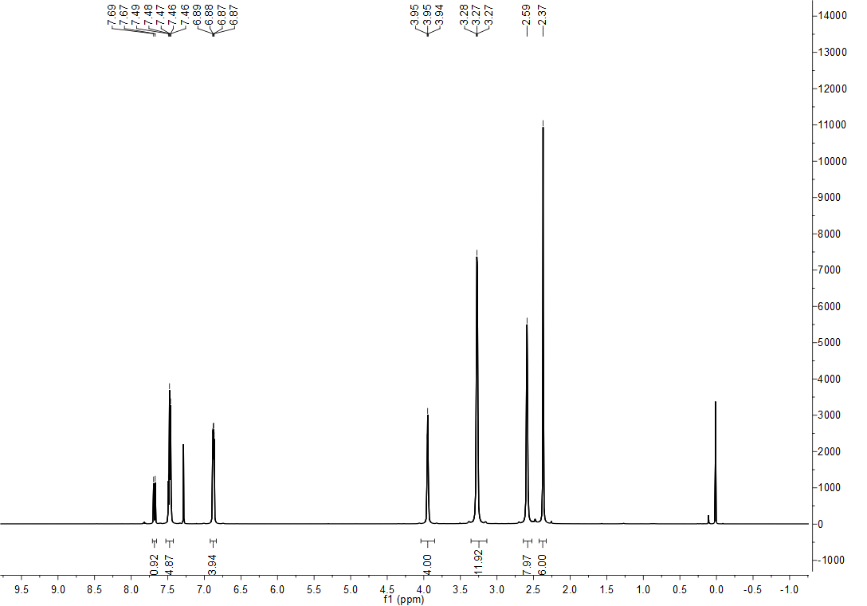


**Figure S32.** ^1^H NMR spectrum of **QN-6**


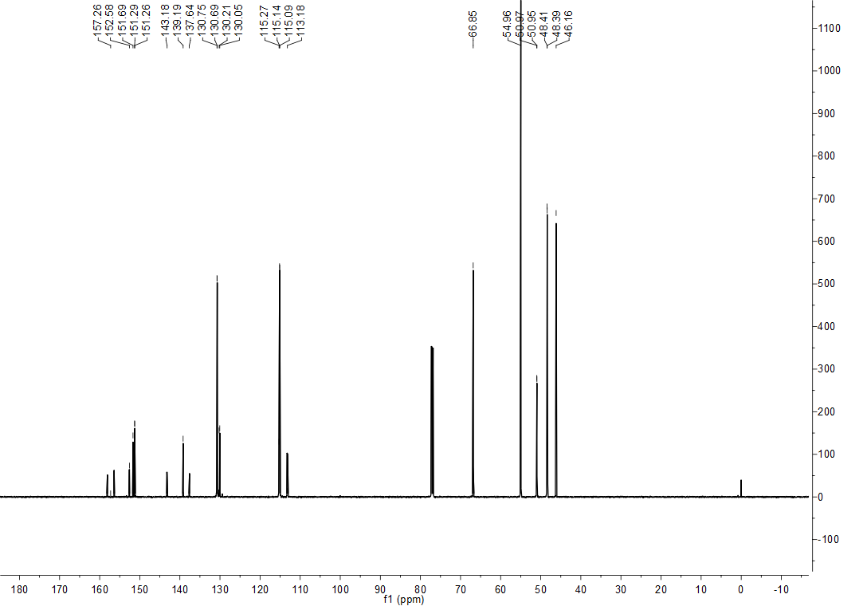


**Figure S33.** ^13^C NMR spectrum of **QN-6**

**Figure S34.** HRMS spectrum of **QN-6**


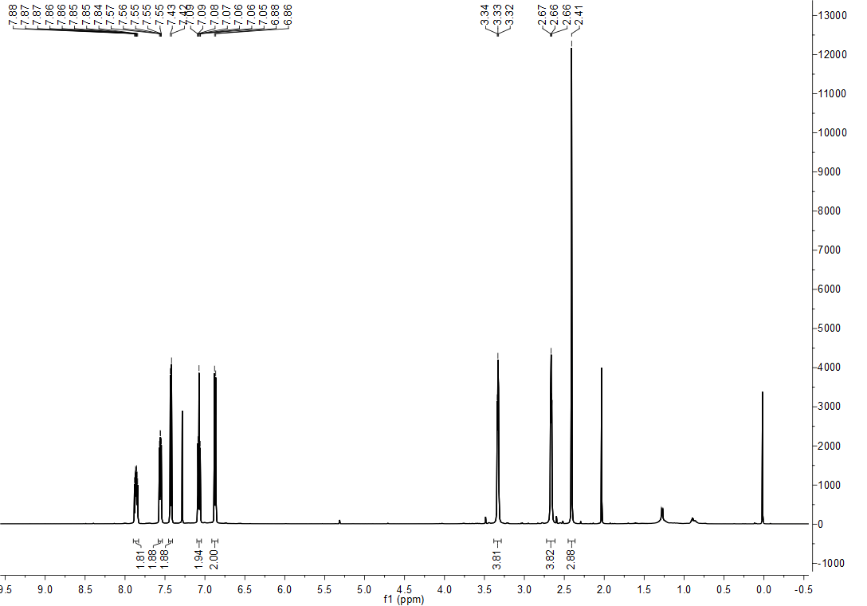


**Figure S35.** ^1^H NMR spectrum of **QN-7**


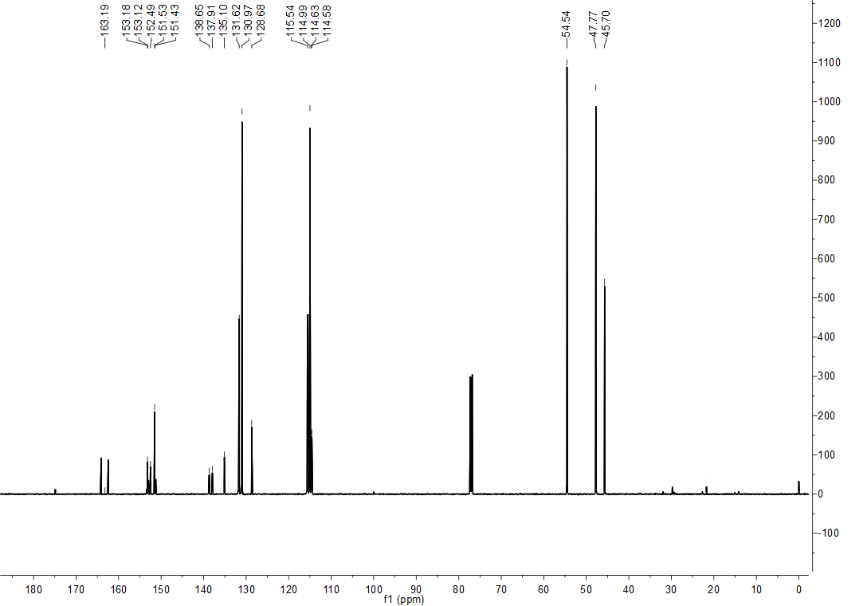


**Figure S36.** ^13^C NMR spectrum of **QN-7**

**Figure S37.** HRMS spectrum of **QN-7**


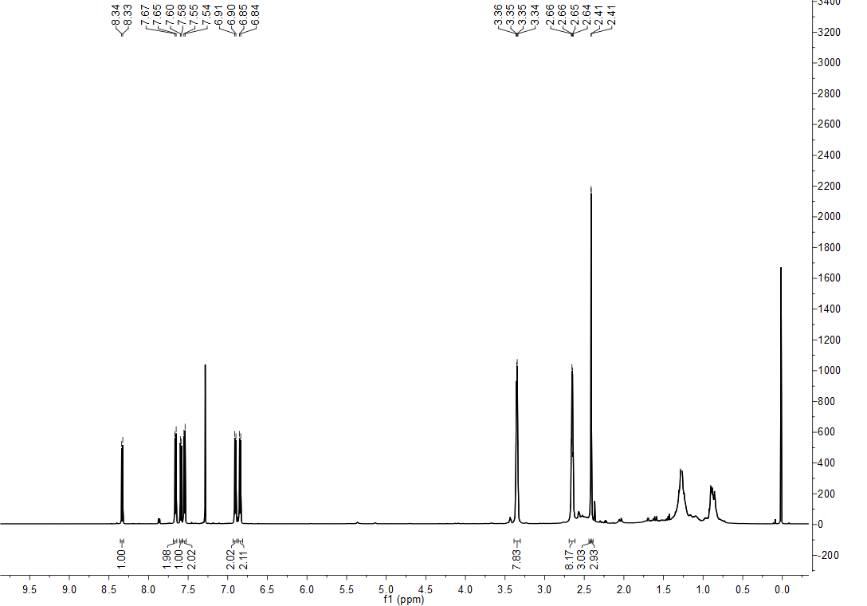


**Figure S38.** ^1^H NMR spectrum of **QN-8**


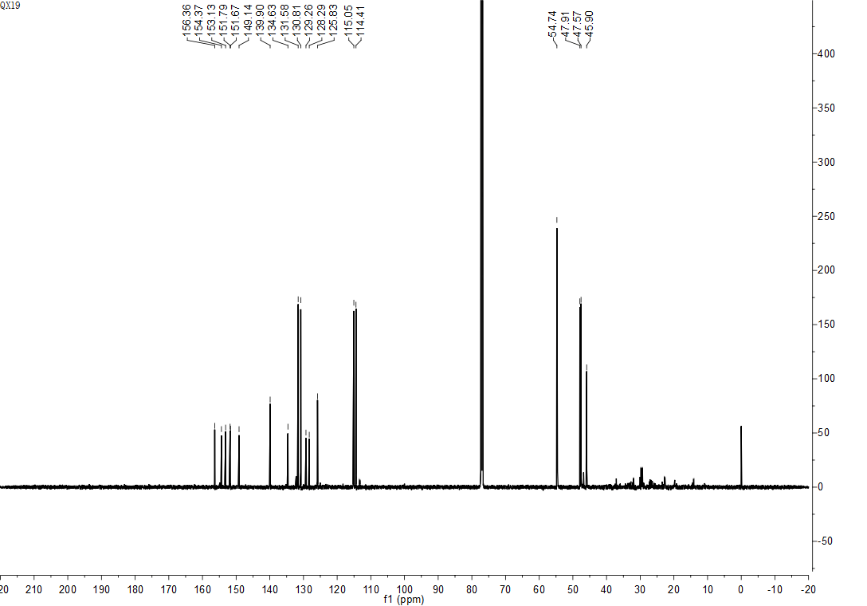


**Figure S39.** ^13^C NMR spectrum of **QN-8**

**Figure S40.** HRMS spectrum of **QN-8**


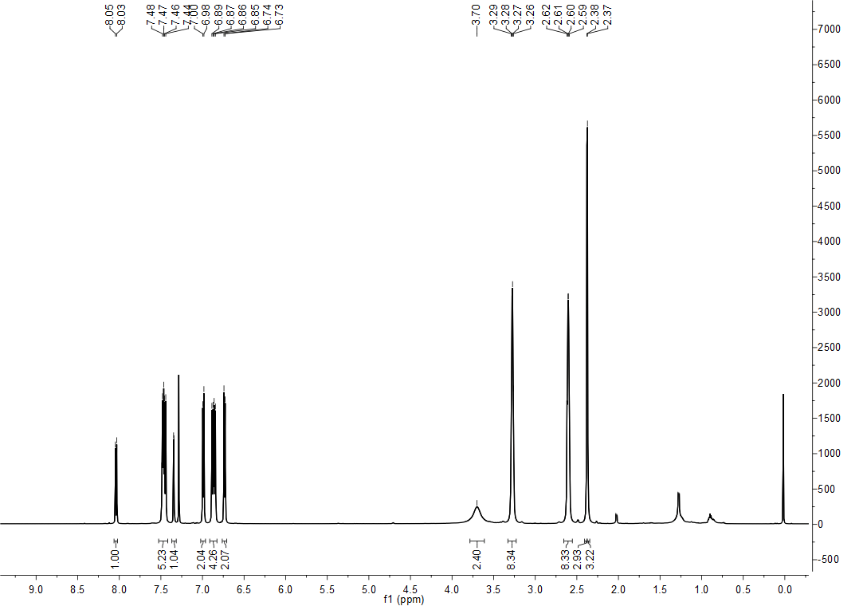


**Figure S41.** ^1^H NMR spectrum of **QN-9**


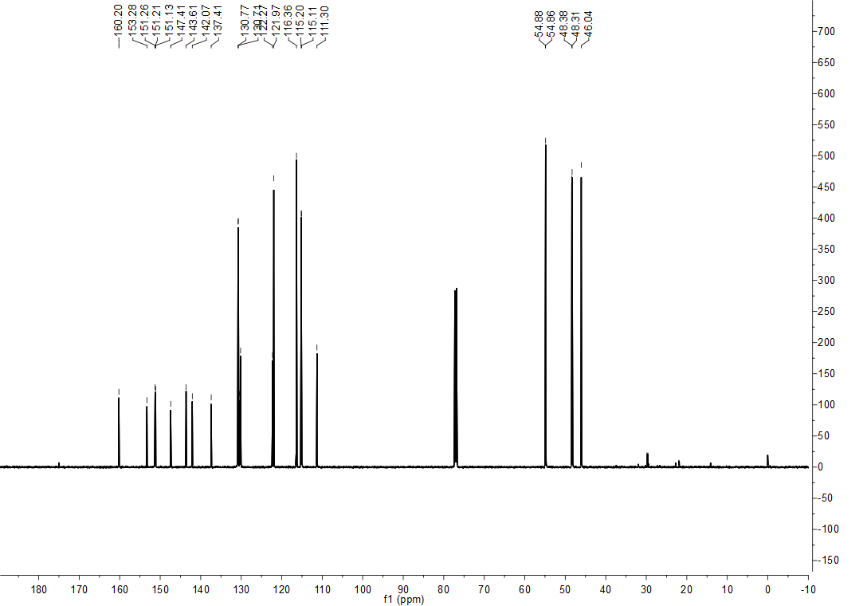


**Figure S42.** ^13^C NMR spectrum of **QN-9**

**Figure S43.** HRMS spectrum of **QN-9**


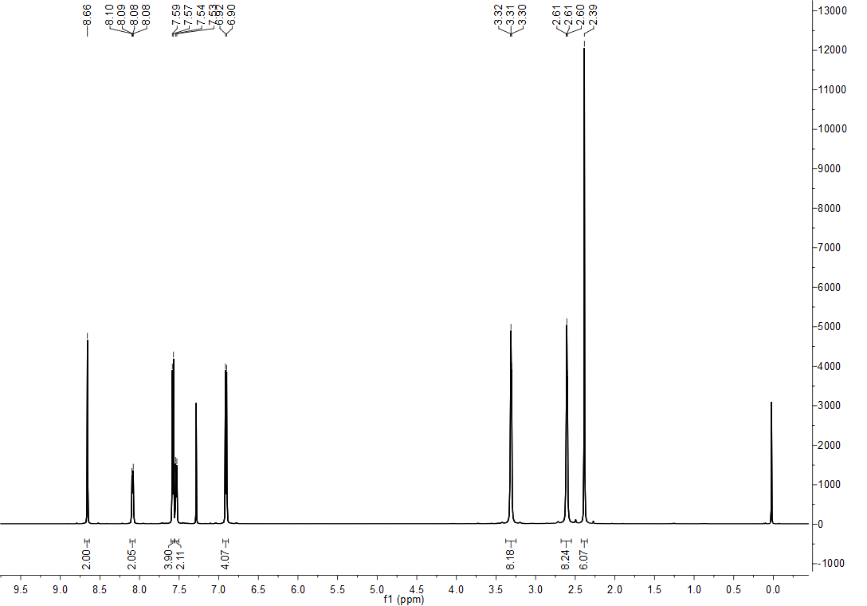


**Figure S44.** ^1^H NMR spectrum of **QN-10**


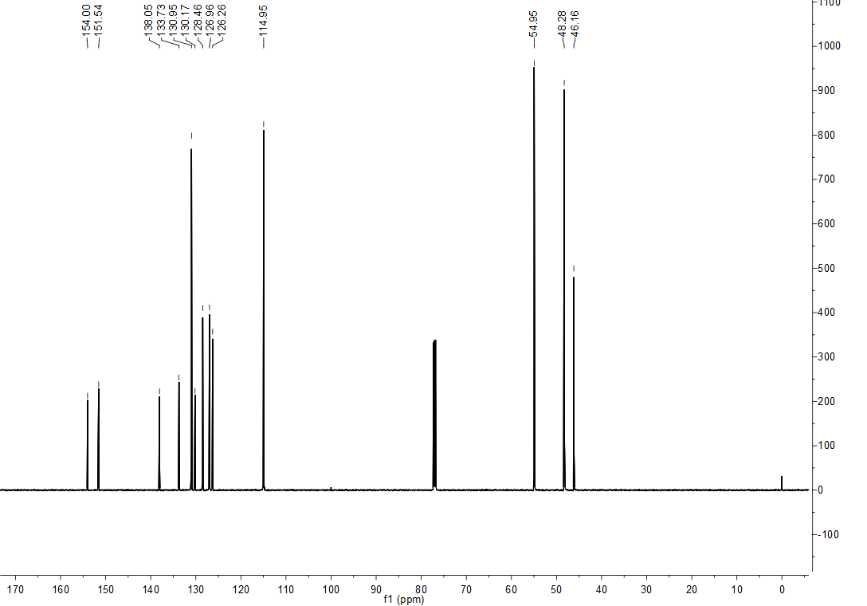


**Figure S45.** ^13^C NMR spectrum of **QN-10**

**Figure S46.** HRMS spectrum of **QN-10**
